# The core domain of hepatitis C virus glycoprotein E2 generates potent cross‐neutralizing antibodies in guinea pigs

**DOI:** 10.1002/hep.28989

**Published:** 2017-02-14

**Authors:** Patricia T. Vietheer, Irene Boo, Jun Gu, Kathleen McCaffrey, Stirling Edwards, Catherine Owczarek, Matthew P. Hardy, Louis Fabri, Rob J. Center, Pantelis Poumbourios, Heidi E. Drummer

**Affiliations:** ^1^ Centre for Biomedical ResearchBurnet InstituteMelbourneAustralia; ^2^ Department of Microbiology and Immunology at The Peter Doherty Institute for Infection and ImmunityUniversity of MelbourneParkvilleAustralia; ^3^ CSL LimitedParkvilleAustralia; ^4^ Department of MicrobiologyMonash UniversityClaytonAustralia

## Abstract

A vaccine that prevents hepatitis C virus (HCV) infection is urgently needed to support an emerging global elimination program. However, vaccine development has been confounded because of HCV's high degree of antigenic variability and the preferential induction of type‐specific immune responses with limited potency against heterologous viral strains and genotypes. We showed previously that deletion of the three variable regions from the E2 receptor‐binding domain (Δ123) increases the ability of human broadly neutralizing antibodies (bNAbs) to inhibit E2‐CD81 receptor interactions, suggesting improved bNAb epitope exposure. In this study, the immunogenicity of Δ123 was examined. We show that high‐molecular‐weight forms of Δ123 elicit distinct antibody specificities with potent and broad neutralizing activity against all seven HCV genotypes. Antibody competition studies revealed that immune sera raised to high‐molecular‐weight Δ123 was poly specific, given that it inhibited the binding of human bNAbs directed to three major neutralization epitopes on E2. By contrast, the immune sera raised to monomeric Δ123 predominantly blocked the binding of a non‐neutralizing antibody to Δ123, while having reduced ability to block bNAb binding to E2, and neutralization was largely toward the homologous genotype. This increased ability of oligomeric Δ123 to generate bNAbs correlates with occlusion of the non‐neutralizing face of E2 in this glycoprotein form. *Conclusion*: The results from this study reveal new information on the antigenic and immunogenic potential of E2‐based immunogens and provide a pathway for the development of a simple, recombinant protein‐based prophylactic vaccine for HCV with potential for universal protection. (Hepatology 2017;65:1117‐1131).

**Abbreviations**
Δ123: E2‐receptor‐binding domain lacking HVR1, HVR2, and igVR
bNAbs: broadly neutralizing antibodies
G: genotype
DAA: direct‐acting antiviral
ELISA: enzyme‐linked immunosorbent assay
HCV: hepatitis C virus
HCVcc: cell‐culture–derived HCV
HCVpp: HCV pseudoparticles
HMW: high molecular weight
HVR: hypervariable region
ID_50_: 50% inhibitory dose
ID_80_: 80% inhibitory dose
IgG: immunoglobulin G
igVR: intergenotypic variable region
MAbs: monoclonal antibodies
MALS: multiangle light scattering
Nabs: neutralizing antibodies
NMAb neutralizing monoclonal antibody: igVR or VR3 intergenotypic variable region
non‐Nabs: non‐neutralizing antibodies
SDS‐PAGE: sodium dodecyl sulfate polyacrylamide gel electrophoresis
SEC: size exclusion chromatography
WT: wild type

Hepatitis C virus (HCV) is a highly variable pathogen that chronically infects 3% of the world's population. HCV circulates as seven highly divergent genotypes (G1‐G7) and over 67 subtypes (a, b, c, etc.), for which no preventative vaccine is available.^1^ Recently, direct acting antivirals (DAAs) have enabled viral clearance to be achieved in >95% of treated individuals. However, DAAs cannot prevent reinfection and their high cost will place a major economic burden on health care systems. Furthermore, an estimated 50 million people have undiagnosed infections providing a means for continued viral spread. A prophylactic vaccine would prevent new infections, and possibly reinfections, and significantly augment elimination programs involving the use of DAAs.^2^ A prophylactic vaccine has therefore been recognized by the World Health Organization as a priority for development.

A major component of the protective efficacy of human viral vaccines is the induction of neutralizing antibodies (NAbs).^3^ In the case of highly variable pathogens such as HCV, NAbs must be highly cross‐reactive so that they can afford protection against the antigenic diversity present in circulating strains. The surface glycoprotein, E2, attaches virions to the cellullar receptor, CD81, and generates both type‐specific and broadly reactive neutralizing antibodies (bNAbs).^4^, ^5^ In natural infection, 30% of individuals spontaneously clear infection and this has been correlated with rapid induction of bNAb and broadly reactive T cells.^6^ In addition, cocktails of broadly neutralizing monoclonal antibodies (NMAbs) can prevent and clear established HCV infection in small animal models of HCV.^7^ However, previous vaccination studies conducted in animals using full‐length or truncated forms of E2, and a phase I clinical trial of a recombinant HCV glycoprotein vaccine elicited limited cross‐genotype neutralization.^8^, ^9^, ^10^, ^11^, ^12^ Like the glycoproteins of other viruses that have evolved advanced immune evasion mechanisms, such as human immunodeficiency virus 1 and influenza virus, HCV E2 is also highly glycosylated and undergoes rapid sequence change in multiple variable regions.^13^, ^14^, ^15^, ^16^, ^17^ A major goal of HCV vaccine development is to produce an immunogen that focuses the immune response on highly conserved NAb epitopes that mediate pan‐genotype neutralization, while minimizing the production of non‐neutralizing antibodies (non‐NAbs) and type‐specific NAbs.

Within E2 are three variable regions. The N‐terminal hypervariable region (HVR) 1 (HVR1) elicits type‐specific NAbs that drive immune escape, occludes NAb epitopes, and confers resistance to neutralization by chronic immune sera and NMAbs.^16^, ^18^, ^19^ HVR2 appears to be in a surface‐exposed flexible region, and the intergenotypic variable region (igVR or VR3) is located within a disulfide‐constrained loop and is also highly flexible.^20^, ^21^, ^22^ Deletion of all three variable regions from a recombinant subdomain of E2 (residues 384‐661) results in the expression of a soluble E2 core domain (Δ123; Fig. 1A) that retains CD81 binding and is recognized by conformation‐dependent human monoclonal antibodies (MAbs).^23^ The three variable regions modulate the ability of both NAbs and non‐NAbs to recognize their epitopes and so may function to modulate the NAb response to native E2 immunogens.^17^ In this study, we examined whether variable region removal alters the breadth and specificity of the antibody response to E2 and how the inherent properties of E2 to form high‐order oligomers affects its antigenicity and immunogenicity.

**Figure 1:**
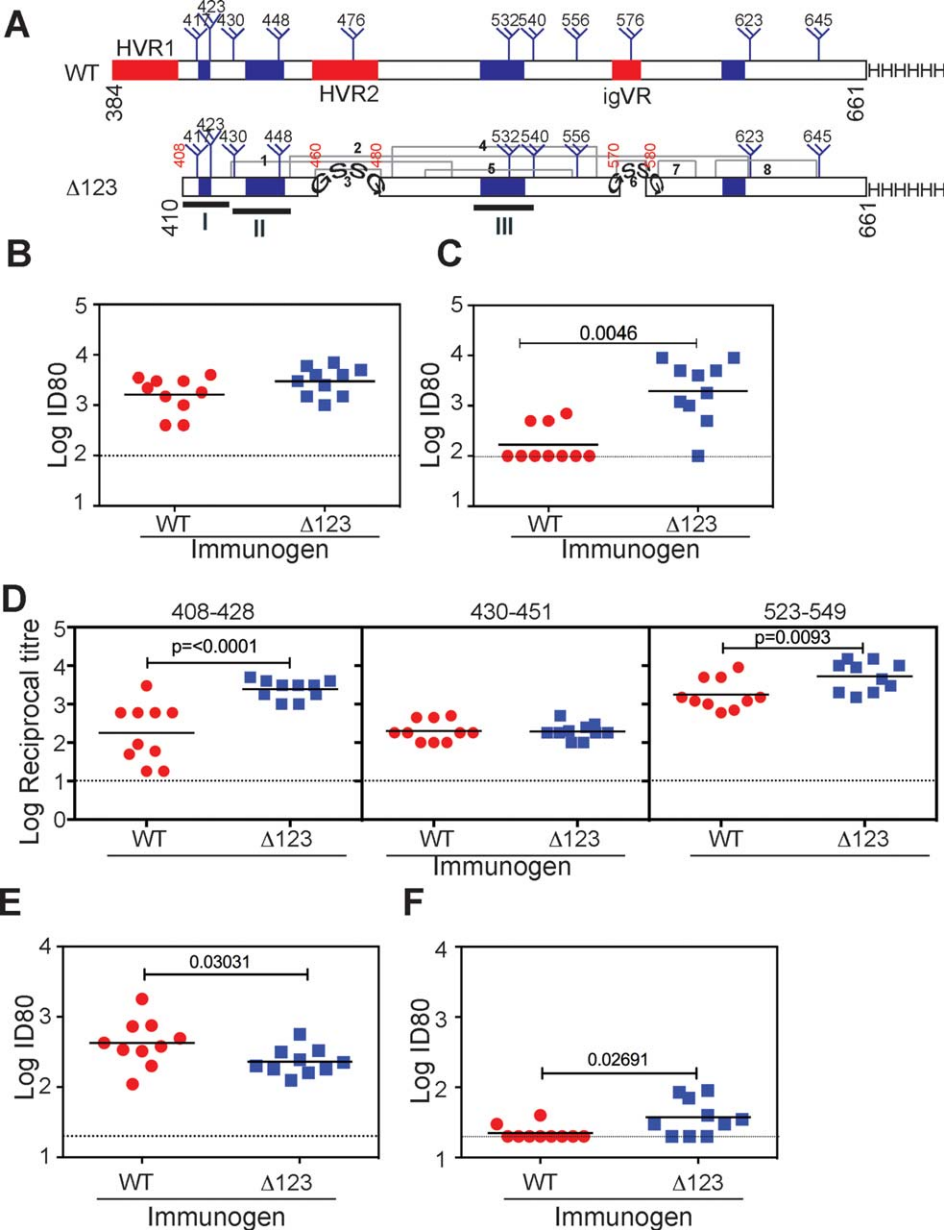
Immunogenicity of affinity‐purified WT and Δ123 E2 proteins in guinea pigs. (A) Schematic of G1a WT (residues 384‐661) and Δ123 E2 showing the location of glycosylation sites (trees), variable regions (red), and Gly‐Ser‐Ser‐Gly linkers that were used to replace the variable regions. Regions corresponding to amino acid residues 408‐428, 430‐451, and 523‐549 are underlined and the C‐terminal poly‐His tag is shown as HHHHHH. Gray lines indicate disulfide linkages observed in Ref 21. (B) Ability of immune sera to inhibit the binding of solution‐phase homologous WT E2 to solid‐phase recombinant CD81‐LEL in ELISA. (C) Ability of immune sera to inhibit the binding of heterologous genotype 2a JFH1 WT E2 protein to recombinant CD81‐LEL. (D) Ability of immune sera to bind synthetic G1a peptides 408‐428, 430‐451, and 523‐549 in ELISA. (E) Ability of immune sera to neutralize homologous G1a HCVpp. The data were derived from three independent experiments performed in triplicate. (F) Ability of immune sera to neutralize a heterologous G2a HCVcc virus. The data were derived from two independent experiments performed in triplicate. The mean level of background inhibition (B,C), binding (D), or neutralization (E,F) achieved by the no‐antigen control group is shown as a dotted line. Horizontal bars represent the geometric mean. *P* values were determined using the Mann‐Whitney *t* test (Prism v 6.0f).

## Materials and Methods

### EXPRESSION VECTORS AND PLASMIDS

The convention of H77 numbering is used throughout. Details can be found in the Supporting Information.

### PROTEIN EXPRESSION AND PURIFICATION

Transient transfections of FS293F cells with the HCV H77c wild‐type (WT) E2 and Δ123 expression plasmids were performed using 293fectin (Invitrogen, Thermo Fisher Scientific, Waltham, MA, USA.) according to the manufacturer's recommendations. Culture supernatants containing secreted glycoproteins were filtered, affinity purified, and then subjected to size exclusion chromatography (SEC) on Superdex 200 26/60 columns (GE Healthcare, Little Chalfont, UK.) equilibrated in PBS (pH 6.8) and fractions pooled based on analytical size exclusion analysis. Dimeric CD81‐LEL was expressed and purified as described.^24^

### ANIMAL ETHICS AND IMMUNIZATIONS

All animal experiments were performed under the Australian code of practice for the care and use of animals for scientific purposes 7th edition under CSL Limited Animal Ethics Committee approval number #771‐3. Groups of 8 age‐matched Albino Dunkin Hartley guinea pigs were used and their weights ranged from 340 to 370 g at first dose. Details of the immunization procedure can be found in the Supporting Information.

### DIRECT‐BINDING ENZYME‐LINKED IMMUNOSORBENT ASSAYS

Direct‐binding enzyme‐linked immunosorbent assays (ELISAs) were performed as described,^17^ and antibody titers were calculated as the reciprocal dilution of immune serum that gave at least 5 times background binding. Further details can be found in the Supporting Information.

### NEUTRALIZATION ASSAYS

Production of HCV pseudoparticles (HCVpp) was performed as described previously 25. Infectious cell‐culture–derived HCV (HCVcc) were produced by transfecting Huh7.5 cells with *in vitro* transcribed RNA as described.^26^ Transfection was performed using either DMRIE‐C reagent (Invitrogen, Thermo Fisher Scientific) or by electroporation as described.^26^ Tissue culture fluid collected 72 hours later was filtered through 0.45‐μm syringe filters, concentrated through 100K MWCO Centrifugal Concentrators (Sartorius, Göttingen, Germany), and stored at –80°C. Details of NAb assays are described in the Supporting Information.

### STATISTICAL ANALYSIS

Replicate data were fitted to a curve of best fit using Hills One‐site specific binding plot. For comparison of two groups, an unpaired nonparametric Mann‐Whitney *t* test was applied. For comparison of more than two groups, the Kruskal‐Wallis test with Dunn's posttest correction for multiple comparison was used. Exact *P* values are shown unless *P* < 0.0001. All statistical tests were performed in Prism v 6.0f or 7.0a. Where no value is shown, *P* > 0.05.

## Results

### THE Δ123 IMMUNOGEN ELICITS A DISTINCT ANTIBODY SPECIFICITY COMPARED TO WT IMMUNOGEN

To investigate whether removal of all three variable regions alters the immune response to E2, Hartley Albino guinea pigs were immunized subcutaneously three times with 100 μg of nickel affinity purified WT E2 or Δ123 antigens derived from the G1a strain, H77c (Fig. 1A) in ISCOMATRIX adjuvant. WT‐ and Δ123‐immune sera exhibited similar antibody titers toward both WT and Δ123 antigens (Supporting Fig. S1A,B), and, as expected, only immune sera raised to WT E2 possessed HVR1 peptide‐specific antibodies (Supporting Fig. S1C).

A feature of a number of NMAbs is their ability to prevent binding of E2 to CD81. We used a previously characterized E2‐CD81 binding assay to examine whether immune sera could block this interaction.^24^ Both WT and Δ123 immune sera prevented the interaction between homologous G1a E2 and the CD81 large extracellular loop (CD81‐LEL; Fig. 1B). However, animals vaccinated with Δ123 generated significantly higher 80% inhibitory titers (80% inhibitory dose; ID_80_) against heterologous G2a E2 compared to the WT vaccinated group (*P* = 0.0046; Fig. 1C). The specificities of antibodies generated by Δ123 and WT were explored in ELISA employing three homologous G1a synthetic peptides that encompass or overlap epitopes of human NMAbs to HCV and contain CD81‐binding residues.^17^, ^21^, ^22^ Peptide 408‐428 is a target of NMAb HCV1 isolated from human immunoglobulin G (IgG) transgenic mice and human NMAb HC33.1, and rodent NMAbs MAb24 AP33, and 3/11, and contains two CD81 contact residues, W420 and H421.^17^, ^27^, ^28^, ^29^, ^30^ Peptide 430‐451, is a target of human NMAbs HC84‐1 and HC84‐27^31^ and contains CD81 contact residues N430, G436, W437, L438, G440, L441, F442, and Y443, whereas peptide 523‐549 contains CD81‐binding residues G523, Y527, W529, G530, D535, N540, and W549^5^, ^17^, ^21^ (Table 1). The results show that Δ123 elicited significantly higher antibody titers to peptides 408‐428 and, to a lesser degree, to peptide 523‐549 (*P* = 0.0093) when compared to WT E2 immune sera (Fig. 1D).

**Table 1:** Relative Binding of MAbs to Oligomeric Δ123

MAb	NAb activity^a^	Epitope type^b^	Residues known to affect binding^c^	CD81 blockade^d^	HMW1^e^	HMW2^e^	Dimer^e^
HC33.1	Yes	Lin	L413, G418, W420,	+	0.83	2.00	1.50
HC84‐26	Yes	DC	C429, L441, F442, K446, W616,	+	0.48	1.10	1.20
HC‐11	Yes	DC	S424, T425, A426, L427, N428, C429, T435, G436, W437, L438, F442, Y443, K446,Y527A, W529, G530,D535, V536	+	<0.11	0.37	0.67
HC84‐22	Yes	DC	W420, I422, A426, L427, N428, C429,W437, L441, F442, Y443, G530, D535, W616	+	0.13	0.42	0.77
HC‐1	Yes	DC	A426, N428, C429, W529, G530, D535	+	0.23	0.45	0.67
CBH‐4D	No	DC	V536, R630‐G635, P612, L615,	–	<0.15	<0.15	<0.15
CBH‐4B	No	DC	R630‐G635	–	<0.02	0.03	0.34
HC84‐1	Yes	DC	A439, L441, F442, Y443, K446	+	0.77	1.30	0.67
HC84‐27	Yes	DC	A439, L441, F442, Y443, Q444, K446, W616	+	1.10	1.40	1.30
CBH‐7	Yes	DC	N540, W549	+	0.36	0.71	0.91
AR1A	No	DC	T416, N417, P484, Y485, V538, **N540**, G547, W549	+	0.43	1.00	1.00
AR1B	No	DC	Q412,W420, N423, R483, P484, Y485, G523, P525, T526, G530, T534, **N540**, P544, P545, G547, W549	–	1.00	1.00	1.00
AR2A	Yes	DC	**N540**	–	1.00	1.00	1.00
AR3A	Yes	DC	S424, G523, P525, G530, D535, V538, **N540**	+	0.20	0.50	1.00
AR3B	Yes	DC	Q412, T416, G418, N423, S424, G523, P525, G530, D535, **N540**	+	0.01	0.13	0.42
AR3C	Yes	DC	I422, T425, L427, C429, N430, E431, S432, L433, G436, L438, A439, L441, F442, Y443, K446, W529	+	0.28	0.71	1.00
AR3D	Yes	DC	Q412, S424, G523, G530, D535	+	0.03	0.24	0.63
HCV1	Yes	Lin	L413, N415, G418, W420, I422	+	1.00	1.00	1.00
H53	No	DC	N540, W549	–	1.00	1.00	1.00
H52	No	Lin	C652	–	3.50	3.50	3.50
MAb24	Yes	Lin	L413, I414, N415, T416, G418, W420, H421	+	1.10	1.80	1.70
MAb44	Yes	Lin	G523, P525, **N540**, W549, Y613	+	0.91	1.00	1.00
MAb26	No	Lin	N645‐E661	–	1.00	1.00	1.00
MAb6	No	Lin	Y527, W529, G530, D535	–	0.59	1.00	1.00
MAb13	No	Lin	Y527, W529, G530	–	0.71	1.40	1.10
MAb25	No	Lin	Y527, W529, G530. D535	–	0.77	1.10	1.00
MAb14	No	DC	P525, Y527, W529, G530	–	0.56	1.00	0.91
MAb22	No	Lin	Y527, W529, G530	–	0.50	1.00	0.91
MAb39	No	Lin	G523, P525	+	0.77	0.83	0.83

a Demonstrated ability to neutralize at least homologous virus.

b Epitope designated as discontinuous (DC) when binding is dependent on E2 fold or Linear (Lin) when the MAb binds denatured antigen or a synthetic peptide.

c Amino acid residues known to affect binding of MAb by at least 50%. References can be found in Supporting Table S2. For MAbs where crystal structures are available (HC84‐1, HC84‐27, HCV1, and AR3C), residues buried by more than 10Å are listed. Bold residue is the site of an N‐linked glycan. Residues implicated in CBH‐4B and CBH‐4D binding are a personal communication from Steven Foung. Epitope for H52 is an unpublished observation (H.E. Drummer).

d Capacity of MAb to block interaction between E2 and CD81.

e Relative binding of MAbs to oligomeric forms of Δ123 relative to monomeric Δ123. Original data shown in Supporting Fig. S4.

The ability of immune sera to neutralize viral entry was next examined. Animals vaccinated with WT E2 antigen elicited slightly higher homologous HCVpp NAb titers compared to the Δ123 immunogen (*P* = 0.03031; Fig. 1E) that may reflect the presence of NAbs directed to HVR1 in WT immune sera (Supporting Fig. S1C). The ability of immune sera to neutralize entry and replication of the Jc1Flag2 (p7‐nsGluc2A) HCVcc, which encodes the G2a structural proteins of HCV J6, was examined.^32^ H77c and J6 E2 (residues 384‐661) differ by 29% (Supporting Table S1; Supporting Fig. S2). Whereas overall ID_80_ titers were modest, higher titers of G2a HCVcc NAbs were generated in animals vaccinated with Δ123 compared to WT vaccinated animals (*P* = 0.02691). The number of animals generating cross‐reactive NAbs was also found to be higher in the Δ123 group (7 of 10 for Δ123 versus 2 of 10 for WT), with the mean ID_80_ titer being 2.6 ± 1.4 fold higher compared to the WT group (Fig. 1F). These data suggest that NAbs present in WT immune sera are focused on type‐specific epitopes in HVR1 with type‐specific E2‐CD81 blocking ability and limited cross‐neutralization activity. By contrast, Δ123 elicited higher antibody titers to peptides 408‐428 and 523‐549; the antibodies were able to cross‐inhibit G2a E2‐CD81 binding with a higher capacity to cross‐neutralize G2a HCVcc. These results indicate that Δ123 has favorable immunogenic properties.

### PURIFICATION OF OLIGOMERIC FORMS OF Δ123 AND THEIR ANTIGENICITY

**Figure 2:**
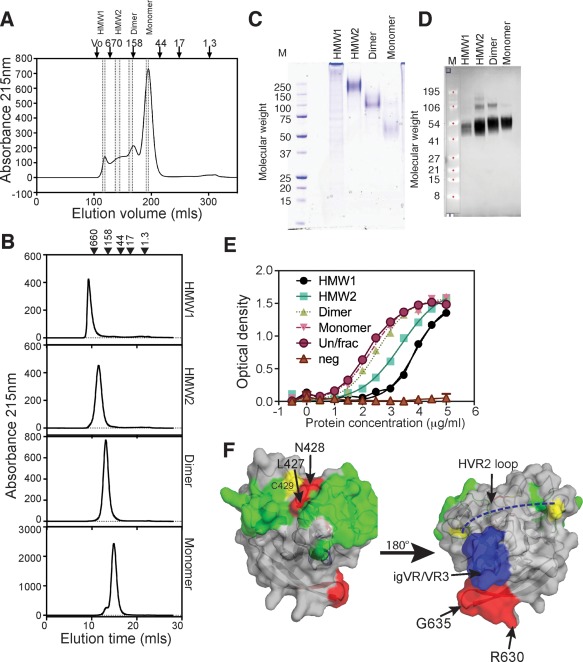
SEC purification and characterization of Δ123 species. (A) SEC profile of Δ123 using Superdex 200 showing areas of the profile that were pooled to generate HMW1, HMW2, dimer, and monomer species (area between dotted lines). The peak elution positions of standard proteins in kDa are shown above the chromatogram. (B) SEC profiles of individual purified species. (C) Nonreducing SDS‐PAGE and Coomassie blue staining of purified proteins (3 μg). Markers are shown on the left (kDa). (D) Western blotting of reduced proteins transferred to nitrocellulose membrane and detected with anti‐His antibody and scanned on an Odyssey imager. Markers are shown on the left (kDa). (E) Ability of proteins to directly bind dimeric CD81‐LEL. No binding was observed to the F186S CD81‐LEL mutant that abrogates E2‐CD81 interactions (neg). (F) Placement of residues that are occluded on the core domain of E2 (derived from Table 1 and Supporting Fig. S4). Red = residues that are occluded in HMW1. Blue surface = igVR/VR3 region. Dashed blue line is the putative location of HVR2. Yellow = Cys residues. Green = residues involved in binding CD81. On the left is the neutralizing face of E2 and on the right is the non‐neutralizing face of E2. N‐linked glycans are not shown. Arg630‐Gly635 is shown in red.

To further understand the biochemistry, antigenic, and immunogenic properties of Δ123 E2_,_ SEC and sodium dodecyl sulfate polyacrylamide gel electrophoresis (SDS‐PAGE) analysis was performed. The analyses revealed that the Ni^2+^‐affinity–purified Δ123 preparation comprises a continuum of oligomeric forms ranging in molecular mass from 44 to >600 kDa (Fig. 2A). A similar SEC profile was observed for WT E2, suggesting that the size heterogeneity is not related to variable region deletion (Supporting Fig. S3A). Four representative areas of the SEC fractionation were collected (Fig. 2A) and reanalyzed on SEC to confirm their sizes and homogeneity (Fig. 2B). SEC, followed by multiangle light scattering (MALS), was used to estimate the size of the proteins (Table 2). Two areas of the fractionation were selected to represent high‐molecular‐weight (HMW) forms of Δ123 with molecular masses of 2,402 (high‐molecular‐weight 1; HMW1) and 239.3 kDa (HMW2). Peaks corresponding approximately in molecular mass to dimers (97.3 kDa) and monomers (46.6 kDa) were also collected. Nonreducing SDS‐PAGE confirmed their approximate sizes and revealed greater than 90% purity (Fig. 2C). Each of these species migrated as monomers in reducing SDS‐PAGE, indicating that disulfide linkages contribute to their higher‐order structures (Fig. 2D). Differences in the migration of reduced monomers in SDS‐PAGE are likely attributed to glycosylation differences given that N‐linked glycan removal with PNGaseF treatment converted the E2 species to the backbone molecular mass of 26 kDa (not shown). Although each of these forms could bind CD81‐LEL, binding was inversely proportional to oligomeric valency (Fig. 2E).

**Table 2:** SEC‐MALS Analysis of the Molecular Mass of Different Species of Δ123

Δ123 Species	MW (kDa)	Uncertainty (%)	Polydispersity (MW/Mn)	Uncertainty (%)	Calculated Mass (μg)	Ratio Mono‐ vs. Multimer
Monomer	46.6	0.70	1	0.90	30.21	1.0
Dimer	97.3	0.90	1	1.20	11.79	2.1
HMW2	239.3	0.60	1	0.80	4.01	5.1
HMW1	2,402.4	0.70	1	1.00	0.65	51.6

We examined whether the differences in oligomeric structure were reflected in antigenic differences using a panel of conformation‐sensitive and ‐insensitive MAbs, isolated from HCV‐infected humans^5^, ^33^, ^34^, ^35^, ^36^ or from mice immunized with H77c WT E2 or Δ123,^17^, ^37^ in ELISA. The ability of a subset of MAbs to bind particular Δ123 forms was inversely proportional to the mass (Table 1; Supporting Fig. S4). For example, the human non‐neutralizing MAbs, CBH‐4B and CBH‐4D, and the NMAbs, HC‐11, HC84‐22, HC‐1, AR3A, AR3B, AR3C and AR3D, all showed sequential reductions in their ability to bind dimer, HMW2, and HMW1, whereas the human MAbs, HC33.1, HC84‐26 and AR1A, and mouse MAbs 6, 13, 14, 22, and 25 displayed reduced binding to only HMW1. The location of the MAb epitopes (Supporting Fig. S5) indicate that two regions are inaccessible and/or conformationally altered in HMW1: Ser424‐Asn428 and Arg630‐Gly635^21^, ^22^ (Supporting Fig. S5). Placement of these residues on the three‐dimensional structure of the E2 core domain reveals that the 630‐635 region is located on the non‐neutralizing face of E2, in close proximity to the igVR/VR3 region and HVR2 domains, whereas 424‐428 is in close proximity to Cys429 (Fig. 2F). The effect of Δ123 aggregation on the Ser424‐Asn428 region appears to be highly localized given that the binding of MAbs specific to epitopes located immediately N‐terminal (HC33.1, HCV1, and MAb24) or C‐terminal (HC84‐21 and HC84‐27) to this region were largely unaffected. Whereas non‐NAb epitopes were also occluded, epitopes recognized by NAbs, including HC‐11, HC84‐22, HC‐1, HC84‐1, HC84‐27, AR2A, AR3C, and AR3D, were more occluded in all WT E2 oligomers compared to the corresponding Δ123 antigen (Supporting Table S2). These differences between Δ123 and WT E2 are likely attributed to the presence of the variable regions occluding a larger glycoprotein surface area in WT E2.^17^

### IMMUNOGENICITY OF OLIGOMERIC FORMS OF Δ123

**Figure 3:**
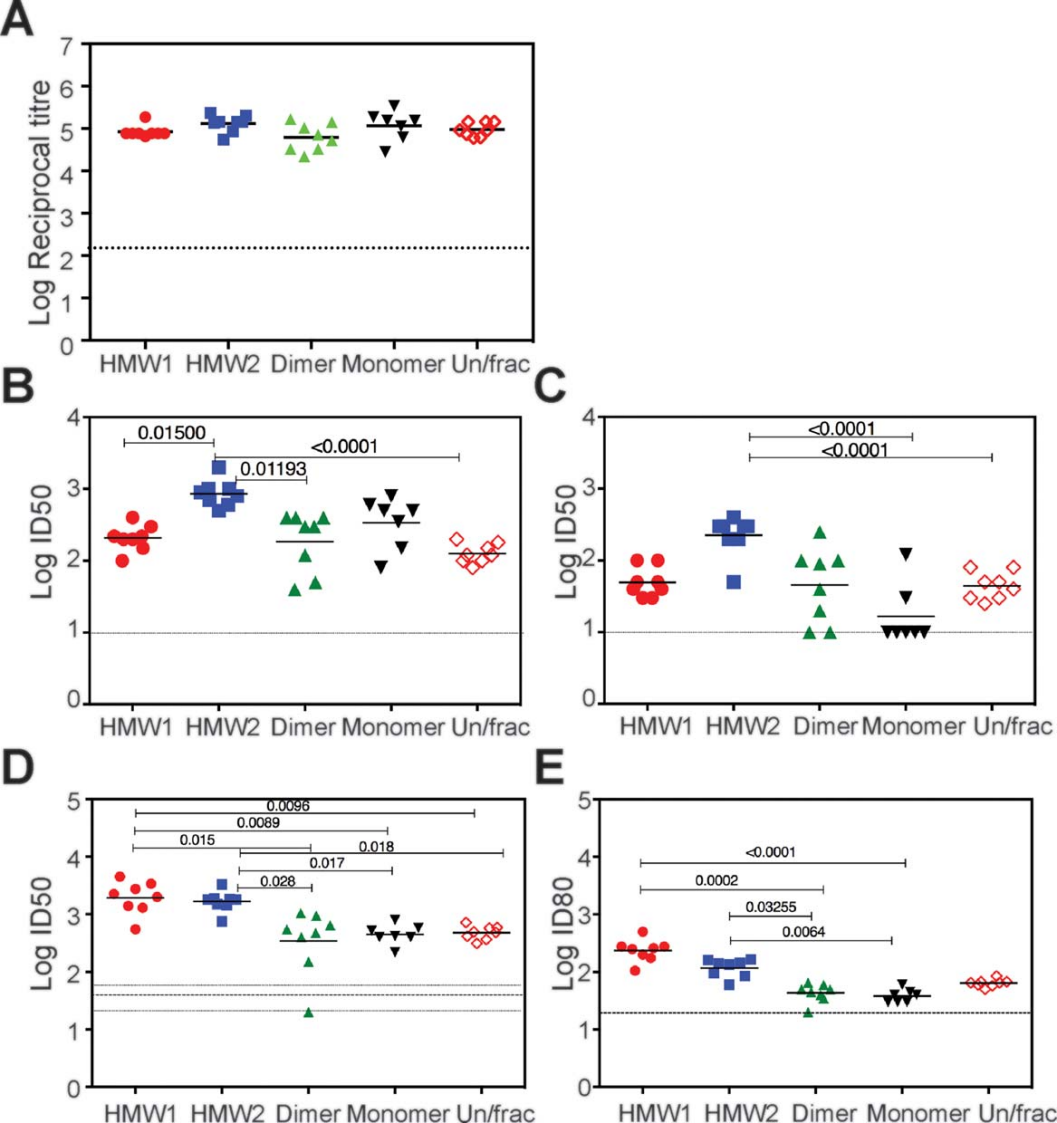
Immunogenicity of SEC fractionated species of Δ123. (A) Reactivity of immune sera toward homologous G1a monomeric Δ123 purified as described in Fig. 2. The affinity‐purified antigen preceding SEC is referred to as Un/frac here and throughout. (B) Ability of immune sera to inhibit the binding of homologous G1a WT E2 protein to recombinant CD81‐LEL (C) Ability of immune sera to inhibit the binding of heterologous G2a JFH1 WT E2 protein to recombinant CD81‐LEL. (D) ID_50_ neutralization titers against homologous HCVpp and (E) ID80 neutralization titers against homologous HCVpp from two independent experiments performed in triplicate. *P* values were determined using the Kruskal‐Wallis test with Dunn's post‐test correction for multiple comparison (Prism v 6.0f). The mean level of background binding (A), E2‐CD81 inhibition (B,C), or neutralization (D,E) achieved by the no‐antigen control group is shown as a dotted line. In (D), the SE above and below the central mean is shown as dotted lines. Horizontal bar is the geometric mean.

The immunogenicity of SEC‐fractionated Δ123 HMW1, HMW2, dimer, and monomer was compared with that of the unfractionated Δ123. All animals elicited similar titers of antibody reactive to monomeric Δ123 (Fig. 3A). Similar homologous E2‐CD81 blocking titers were elicited by HMW1, dimeric, and monomeric Δ123 vaccines, but 50% inhibitory titers (ID_50_) were significantly higher in the HMW2 immune group compared to HMW1, dimer, and unfractionated groups (Fig. 3B). All animals in HMW1, HMW2, and unfractionated immunogen groups achieved ID_50_ titers for heterologous E2‐CD81 binding, with significantly higher inhibitory activity observed for HMW2‐immune sera relative to monomer and unfractionated groups (Fig. 3C). HMW1 sera exhibited similar homologous and heterologous E2‐CD81 ID_50_ titers; however, ID_80_s were not achieved despite having similar overall antibody‐binding titers to the HMW2 immune sera (Supporting Fig. S6A,B). The antibody responses in animals vaccinated with monomeric Δ123 were largely genotype specific in their capacity to inhibit E2‐CD81 interactions (Fig. 3C and Supporting Fig. S6B).

The abilities of the different oligomeric forms of Δ123 to elicit NAbs against homologous G1a HCVpp were determined. HMW1 and HMW2 vaccine groups had significantly higher ID_50_ titers compared to the dimer, monomer, or unfractionated Δ123 groups (Fig. 3D). In addition, the ID_80_s were significantly higher in HMW1 and HMW2 groups compared to both dimer and monomer groups (Fig. 3E). The data suggest that the Δ123 HMW form generates higher titers of homologous NAb.

### HMW1 AND HMW2 IMMUNE SERA ELICIT CROSS‐NEUTRALIZING ANTIBODIES

**Figure 4:**
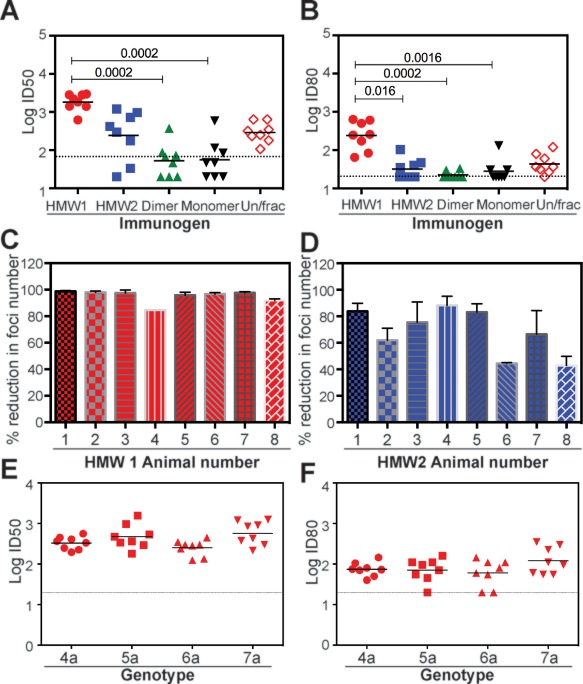
Analysis of the ability of immune sera to cross‐neutralize heterologous genotypes of HCV. (A) ID_50_ neutralization titers against G2a HCVcc virus and (B) ID_80_ neutralization titers against G2a HCVcc virus from three independent experiments performed in triplicate. (C) Ability of HMW1‐immune sera to neutralize a G3a HCVcc virus from two independent experiments performed in duplicate. (D) Ability of HMW2‐immune sera to neutralize a G3a HCVcc virus. For (C) and (D), mean ± SD shown from two experiments performed in triplicate. (E) ID_50_ neutralization titers of HMW1‐immune sera against G4a, G5a, G6a, and G7a HCVcc viruses. (F) ID_80_ neutralization titers of HMW1‐immune sera against G4a, G5a, G6a, and G7a HCVcc viruses. *P* values were determined using the Kruskal‐Wallis test with Dunn's posttest correction for multiple comparison (Prism v 6.0f). For (A), (B), (E), and (F), the horizontal bar is the geometric mean titer and the dotted line is the mean neutralization value for 5 no‐antigen control animals. ID_50_ and ID_80_ data derived from two independent experiments performed in triplicate.

We then examined whether the G1a Δ123 oligomeric species could induce antibody responses able to cross‐neutralize heterologous genotypes of HCV. HMW1 Δ123‐immune sera possessed significantly higher ID_50_s and ID_80_s against heterologous G2a HCVcc relative to dimer‐ and monomer‐immune sera (Fig. 4A,B); HMW1 elicited significantly higher ID_80_ NAb titers than HMW2 and was able to completely neutralize the G2a strain at a 1:40 dilution (*P* = 0.016). The ID_50_ of Δ123‐monomer and dimer immune sera were approximately 30‐ and 4‐fold lower relative to HMW1 and HMW2 immune sera, respectively (Fig. 4A), and the majority failed to achieve 50% or 80% neutralization. That neutralization was mediated by IgG present in HMW1 and HMW2 immune sera was confirmed by protein G‐Sepharose depletion of homologous NAb activity (Supporting Fig. S7). Furthermore, we confirmed that neutralization was mediated by HCV‐specific antibody by showing that pooled sera did not neutralize retroviruses pseudotyped with rhabdovirus vesicular stomatitis virus G glycoprotein (Supporting Fig. S8).

Given that HMW1 and HMW2 vaccine groups possessed high‐titer cross‐neutralizing antibodies toward G2a HCVcc, we further compared the ability of these sera to cross‐neutralize chimeric HCVcc viruses containing the structural regions from G3 to G7. The E1E2 and E2 regions derived from these genotypes differ from the corresponding regions of H77c by 23‐33% (Supporting Fig. S9; Supporting Tables S1 and S3). The ability of HMW1‐ and HMW2‐immune sera to prevent replication of G3a virus was measured in a focus forming assay using a previously described chimeric full‐length HCV clone.^38^ The results show that sera of all animals in the HMW1 group were able to reduce the number of foci of infection by at least 80%, whereas sera from 6 of 8 animals in the HMW2 group effected a >60% reduction (Fig. 4C,D). For G4‐G7, we used previously described chimeric viruses encoding a luciferase reporter.^39^ The reciprocal geometric mean ID_50_ and ID_80_ values for HMW1‐immune sera against G4a, G5a, G6a, and G7a HCVcc ranged from 250 to 570 and 60 to 120, respectively (Fig. 4E,F). However, although HMW2‐immune sera were able to achieve 50% neutralization (Supporting Fig. S10), 80% neutralization was not observed (not shown). The data suggest that the oligomeric status of Δ123 is a major determinant of its immunogenic properties with respect to cross‐neutralization.

### HMW1 AND MONOMERIC Δ123 ELICIT DIFFERENT ANTIBODY SPECIFICITIES

**Figure 5:**
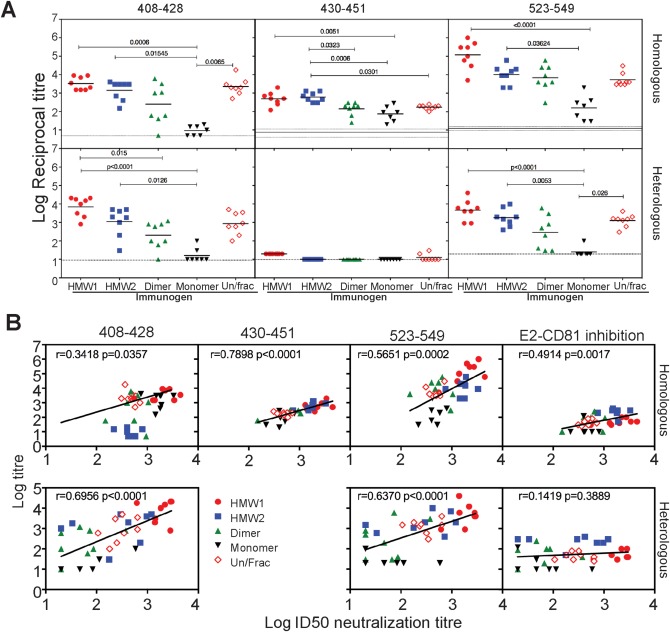
Specificity of the antibody response elicited to different oligomeric forms of Δ123. (A) Ability of immune sera to bind homologous H77c peptides 408‐428, 430‐451, and 523‐549 and heterologous J6 peptides 408‐428, 430‐451, and 523‐549. *P* values were determined using the Kruskal‐Wallis test with Dunn's posttest correction for multiple comparison (Prism v 6.0f). (B) Correlation analysis of homologous and heterologous ID_50_ neutralization titers with homologous and heterologous antibody titers toward peptides 408‐428, 430‐451, and 523‐549 and the ability to inhibit homologous or heterologous E2 binding to CD81. Data were analyzed using Spearman's correlation and the linear regression lines overlaid using Prism (v 7.0a).

We further examined the antibody specificities elicited by the various Δ123 forms using the homologous G1a peptides 408‐428, 430‐451, and 523‐549 and the equivalent heterologous G2a peptide analogs. The results show that HMW1‐ and HMW2‐immune sera had significantly higher titers of antibody reactive to G1a versions of all three peptides compared to monomeric vaccine immune sera (Fig. 5A). When cross‐genotype reactivity was examined, HMW1‐ and HMW2‐immune sera had significantly higher binding titers for G2a peptides 408‐428 and 523‐549 compared to the monomer vaccine group (Fig. 5A). Cross‐reactivity was not observed toward G2a peptide 430‐451. These results reveal that HMW forms of Δ123 elicit cross‐genotype reactive antibodies toward the 408‐428 and 523‐549 regions of E2, whereas monomeric Δ123 does not. We investigated whether antibody specificity to synthetic peptides and/or ability to block E2‐CD81 binding correlated with homologous neutralization of G1a virus and cross‐neutralization of G2a virus. The results show that homologous NAb responses correlated most strongly with reactivity to the 430‐451 peptide (*P* < 0.001) for all immune groups (Fig. 5B). By contrast, cross‐genotype neutralization was most strongly correlated with cross‐reactivity to the G2a peptides representing the 408‐428 and 523‐549 regions (*P* < 0.0001; Fig. 5B).

**Figure 6:**
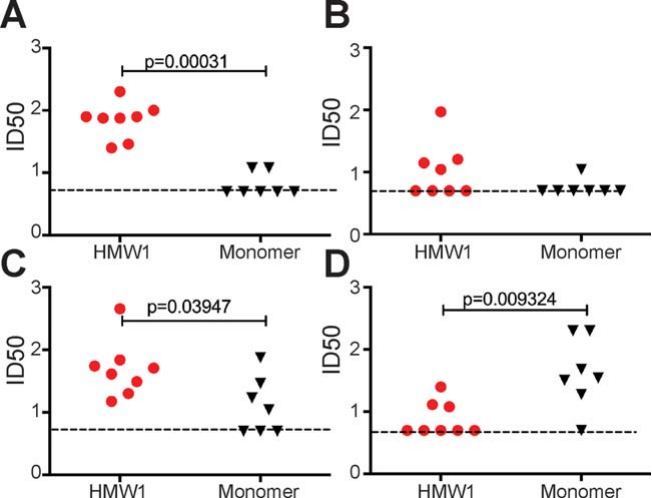
Specificity of HMW1‐ and monomer‐immune sera. Serial dilutions of guinea pig sera were added to a constant amount of HCV1 (A), HC84‐27 (B), AR3C (C), and CBH‐4B (D). Antibodies were added to monomeric Δ123, and bound MAb was detected with anti‐human Fab_2_. The dotted line represents the limit of detection of the assay. Groups were compared using a Mann‐Whitney *t* test (Prism v 7.0a).

To further investigate differences in antibody specificity in HMW1 and monomer immunization groups, we determined the ability of serially diluted immune sera to compete with various neutralizing or non‐neutralizing human MAbs for binding to plate‐bound monomeric Δ123. Binding of MAb to Δ123 should only occur if guinea pig antibodies fail to bind in the vicinity of the MAb epitope. The results indicate that HMW1‐immune sera possessed higher levels of antibodies able to block Δ123 binding by NMAb HCV1 that recognizes its epitope in the context of peptide 408‐428 (Fig. 6A and Supporting Fig. S11) and AR3C (Fig. 6C and Supporting Fig. S11) shown to bind residues within both peptides 430‐451 and 523‐549,^21^ relative to monomer‐immune sera. There was no significant difference in the ability of the immune sera from HMW1‐ and monomer‐immune groups to block binding of HC84‐27 directed principally toward peptide 430‐451 (Fig. 6B and Supporting Fig. S11). By contrast, the non‐neutralizing human Mab, CBH‐4B, directed to the 630‐635 region (Steven Foung, personal communication), was preferentially blocked by monomer‐immune sera (Fig. 6D and Supporting Fig. S11). These data suggest that the preferential induction of bNAbs by HMW1 may depend on the presentation of bNAb epitopes in a context where non‐neutralizing epitopes are occluded (Table 1; Fig. 2F and Supporting Fig. S5). By contrast, the induction of non‐NAbs appears to be favored when the immunogen is monomeric Δ123, which exposes both neutralizing and non‐neutralizing epitopes.

## Discussion

Our studies indicate that the oligomeric state of Δ123 affects its immunogenicity with HMW forms preferentially generating potent bNAbs against the seven genotypes of HCV in a small animal. The findings provide a pathway for the development of a simple, recombinant protein‐based prophylactic vaccine for HCV with potential to provide universal protection.

The Δ123 protein preparation is predominantly comprised of monomers, together with a continuum of oligomeric species, ranging in size from dimers to HMW species containing up to 50 protomers of E2. Four representative species purified from this heterogeneous mixture exhibited distinct antigenic characteristics and immunogenic potentials. One striking feature of the Δ123 oligomers is a progressive decrease in reactivity with the non‐Nabs, CBH‐4B and CBH‐4D, as the number of E2 protomers increased, suggesting that their epitopes (which include residues 630‐635; Steven Foung, personal communication), located on the non‐neutralizing face of E2, become progressively more occluded in oligomeric forms of E2. By contrast, conserved neutralization epitopes overlapping with CD81‐binding sequences located in the 412‐421 region (bNAbs HC33.1, HCV1, and MAb24), the 436‐446 region (bNAbs HC84‐1, HC84‐26, and HC84‐27), and the 523‐540 region (bNAb AR2A, and MAb44), which are located on the opposing face of E2, appear to be similarly exposed on monomer and on oligomeric HMW Δ123 forms. On WT E2 immunogens, non‐NAb epitopes are also occluded. However, compared to Δ123, a number of additional epitopes recognized by NAbs are progressively occluded in dimer and higher‐order species, likely to be a result of the presence of the three variable regions.^17^ These antigenic differences were reflected in the different immunogenic potentials of monomeric Δ123 versus the higher‐order species, with HMW1 Δ123 generating lower titers of non‐NAb antibody specificities and higher titers of antibodies toward two major bNAb epitopes. An examination of the different immunogenic potentials in the context of WT E2 oligomers revealed that high titers of HVR1‐specific antibodies were generated in all groups, despite its occlusion in the WT HMW1 antigen. However, only homologous genotype‐specific E2‐CD81‐blocking antibodies were generated (Supporting Fig. S3D,E), which was concordant with largely homologous neutralization (Supporting Fig. S3F,G). The overall trend that higher‐order forms of E2 are associated with improved cross‐genotype neutralization was also observed for WT E2 immunogens, but this response was less potent than with Δ123 immunogens.

Antibody competition studies were performed with three NMAbs: HCV1, AR3C, and HC84‐27. Our data revealed that when compared to animals receiving monomeric Δ123, HMW1‐Δ123–vaccinated animals generated significantly higher titers of antibody specificities that overlapped with HCV1 and AR3C and 4 of 8 animals also generated HC84‐27‐like antibodies. The bMAb, HCV1, has been shown previously to prevent HCV infection of chimpanzees and to reduce viral load in chronically infected animals.^40^ Keck et al. showed that the HC84 series of NMAbs that includes HC84‐27 do not generate neutralization escape mutants when HCVcc viruses are passaged in their presence.^33^ In addition, when HC84 NMAbs and NMAbs specific to the 412‐428 region represented by HC33‐like NMAbs were combined, E2‐CD81 blockade and neutralization were enhanced in an additive manner, suggesting that cogeneration of these two antibody specificities is desirable in HCV vaccines. That Δ123 HMW1 can simultaneously generate three such bNAb specificities is a major step forward in the HCV vaccine development pathway. By contrast, the predominant antibody specificity generated by animals vaccinated with monomeric Δ123 overlapped with that of the non‐Nab, CBH‐4B, with low titers of neutralizing AR3C‐like antibodies and absent HCV1‐like specificities. The switch in immunodominance observed for the HMW1 vaccine antigen toward antibody specificities implicated in broad HCV neutralization correlates with the observed breadth of neutralization observed for this immune serum.

HCV1‐like antibodies, including HC33.1, MAb24, 3/11, and AP33, that recognize epitopes in the 413‐421 region of E2 are broadly neutralizing and represent desirable specificities in vaccinal immune sera. In natural infection, HCV1‐like antibodies appear to be infrequently elicited, with only 2.5% of sera obtained from chronically infected humans possessing this specificity.^41^ An explanation for the low frequency of HCV‐1‐like antibody specificity may lie in observations that the immunodominant N‐terminal HVR1 region occludes the CD81‐binding site,^16^ potentially impairing the ability of epitopes located in the 413‐421 region to engage B‐cell receptors. In addition, HVR1 increases the resistance of G2a viruses to NMAbs such as AP33.^16^ An examination of the antibody specificities present in WT E2‐immune sera showed high levels of apparent HCV1‐blocking antibodies in HMW1 sera compared with monomer sera (Supporting Fig S12A); however, this did not correlate with strong cross‐neutralization activity. WT E2‐immune sera had lower titers of mostly type‐specific antibodies to peptides 408‐428, 430‐451, and 523‐549 peptides relative to Δ123‐immune sera (Supporting Fig S12E) and suggests that HCV1‐like antibodies were rarely generated in the former. The presence of the variable regions, particularly HVR1, is likely to restrict the generation of HCV1‐like antibodies and limits the cross‐neutralization activity of immune sera generated to WT E2.

Our findings suggest that additional mechanisms may contribute to the subdominance of the 413‐421 epitopes. Monomeric Δ123, which presents the 413‐421 epitopes to a similar extent as HMW1, does not generate high titers of HCV1‐like antibody, but does produce high titers of non‐NAbs. The data suggest that the non‐neutralizing face of monomeric Δ123, that includes the 630‐635 epitope, may be immunodominant with respect to humoral immunity. This is supported by the findings that occlusion of the 630‐635 epitope in HMW1 enabled the preferential generation of high titers of bNAbs, including those directed to 413‐421 region. In WT HMW1, the presence of HVR2 and the igVR/VR3 of WT E2 further occludes non‐NAb sites and resulted in strong suppression of non‐NAb specificities (Supporting Fig S12D). Taken together, these data suggest that the enhanced immunogenicity of HMW1 Δ123 is attributed to the dual effects of deleting the variable regions and occlusion of non‐NAb regions. Such a scenario has been reported for the HIV‐1 glycoproteins, wherein occlusion of the immunodominant non‐neutralizing face is essential for the induction of NAbs to subdominant epitopes.^42^ This observation is explained, in part, by the preference of germline B‐cell receptors for non‐NAb epitopes rather than epitopes recognized by bNAbs. Furthermore, a recent study has demonstrated that the CD81‐binding site in monomeric forms of E2 is flexible and may contribute to the inability to generate NAbs targeting these regions.^20^ Therefore, occlusion of the non‐neutralizing face of E2, while maintaining exposure of bNAb epitopes and stabilization of the 408‐428 region in HMW antigens, may be a key requirement for prophylactic vaccine design. HMW2 comprises five Δ123 units and is more homogeneous than HMW1. However, the non‐neutralizing face is occluded to a lesser extent in HMW2 relative to HMW1, which may explain the lower titers of bNAbs generated by HMW2.

A previous vaccine trial in humans of recombinant E1/E2 purified from CHO cells demonstrated that NAbs were elicited, albeit infrequently, and at relatively low potency, with only 25‐50% homologous neutralization and heterologous neutralization, ranging from 0%‐75%, to G1b, 2a, 3a, 4a, 5a, and 7a and 100% toward G6a being observed.^9^ Our data suggest that WT E2 has reduced ability to generate antibodies that are reactive to the core domain with cross‐neutralizing potential compared to Δ123. The overall immunodominance of HVR1 is likely to be one mechanism resulting in the generation of largely type‐specific neutralizing immune responses. Deletion of HVR1 has been shown to increase the overall neutralization sensitivity of HCVcc isolates from G1‐G6, and suggests that HVR1 occludes underlying neutralization epitopes.^19^ Our data suggest that removal of all three variable regions and the use of HMW antigens that function to occlude an immunodominant epitope at residues 630‐635, and, possibly, stabilize the E2‐CD81–binding site, significantly enhances antibody cross‐reactivity, with increased titers of HCV1‐ and AR3C‐like specificities and broad neutralization against all seven genotypes of HCV. Our findings suggest that the HMW forms of the Δ123 immunogen may provide a pathway for the development of a universal prophylactic HCV vaccine.

Author names in bold designate shared co‐first authorship.

## Supporting information

Additional Supporting Information may be found at onlinelibrary.wiley.com/doi/10.1002/hep.28989/suppinfo.

Supporting Information
