# Supplementary material for: The core domain of hepatitis C virus glycoprotein E2 generates potent cross‐neutralizing antibodies in guinea pigs
Source: Hepatology. 2017 Feb 14;65(4):1117–31. doi: 10.1002/hep.28989 (PMC5408392; doi:10.1002/hep.28989)
Supplement: Supplementary file 1 — Supporting Information [file HEP-65-1117-s001.pdf]

SUPPPLEMENTAL MATERIALS

The core domain of hepatitis C virus E2 glycoprotein generates potent cross-neutralizing antibodies.

Authors: Patricia Vietheer[1,4], Irene Boo[1], Jun Gu[1,4], Kathleen McCaffrey[1,2], Stirling Edwards[3], Catherine Owczarek[3], Matthew P Hardy[3], Louis Fabri[3], Rob J Center[1,2], Pantelis Poumbourios[1,4], Heidi E Drummer[1,2,4*]

Affiliations:

[1]Centre for Biomedical Research, Burnet Institute, Melbourne, Australia.

[2]Department of Microbiology and Immunology at The Peter Doherty Institute for Infection and Immunity, University of Melbourne, Parkville, Australia

[3]CSL Limited, Parkville, Australia.

[4]Department of Microbiology, Monash University, Clayton, Australia.

*Correspondence to: Heidi Drummer. Centre for Biomedical Research, Burnet Institute, 85 Commercial Road, Melbourne VIC 3004, Australia, Email: heidi.drummer@burnet.edu.au. Tel: +61 3 92822179. Fax: +61 3 92822100

SUPPLEMENTAL METHODS.

Vectors. The human codon optimized DNA sequences encoding the H77c (AF009606, G1a) and JFH1 (AB047639, G2a) E2 receptor binding domains encompassing amino acids 384-661 (wild-type, WT) were synthesized by Geneart® (Invitrogen™, Thermo Fisher Scientific) with a Kozak consensus sequence (GCCACC) immediately upstream of the initiating methionine (+1), an N-terminal human trypsin signal peptide and a C-terminal 6x Histidine-tag fused in-frame. The H77c E2 RBD containing deletions of HVR1+HVR2+igVR (Δ123) were generated using standard PCR-based techniques. Using the H77c E2 WT sequence as a template the region encoding HVR1 (387-408) was deleted, while HVR2 (460-485) and the igVR/VR3 (570-580) were replaced with a Gly-Ser-Ser-Gly amino acid linker sequence. Once each cDNA was completed, it was digested with *Nhe*I and *Xho*I, ligated into pcDNA3.1 (Invitrogen™, Thermo Fisher Scientific) and sequenced using Big Dye Terminator v3.1 chemistry.

Expression vectors for the production of HCVpp incorporating E1E2 heterodimers from G1a were pE1E2H77c (1).

The following reagent was obtained through the NIH AIDS Reagent Program, Division of AIDS, NIAID, NIH pNL4-3.Luc.R^-.E^- from Dr. Nathaniel Landau (2, 3). HCVcc were produced from full length *in vitro* transcribed RNA transfected into human hepatoma Huh 7.5 cells from S52/JFH1(T2701G,A4533C), 4a(ED43)-RLucΔ40, 5a(SA13) -RLucΔ40, 6a(HK6a) -RLucΔ40, and 7a(QC69)- RLucΔ40 (Kind gifts from Professor Jens Bukh) and G2a pJC1FLAG2(p7-NS-GLUC2A) (Kind gift from Professor Charles Rice). The large extracellular loop of CD81 was expressed in *E coli* as a maltose binding protein fusion, MBP-$LEL^{113\text{-}201}$ as previously described (4) for use in enzyme linked immunosorbent assays (ELISA). The following reagents were obtained through the NIH AIDS Reagent Program, Division of AIDS, NIAID, NIH: pHEF-VSVG from Dr. Lung-Ji Chang (5).

Monoclonal antibodies. Human antibodies AR1A, AR1B, AR2A, AR3A, AR3B, AR3C and AR3D were a kind gift from Mansun law, Scripps Institute and have been described (6). CBH-4B, CBH-4D, HC33.1, HC84.26, HC-11, HC84.22 CBH-7 and CBH-23 were a kind gift from Steven Foung (7-9), Stanford University. HCV-1 (10), HC84.1 and HC84.27 (11) were produced by synthesis of the VH and VL regions (Geneart) and cloning into pcDNA3-tPA-LC and pcDNA3-tPA-HC, respectively, for the expression of IgG1 under the direction of a tissue plasminogen activator (tPA) leader. Heavy and light chain encoding plasmids were transfected into FS293F cells for IgG production. Murine MAbs H53 and H52 were kind gifts from Jean Dubuisson and Harry Greenberg (12). Mouse MAbs 1,6, 7,10,12,13,14,16,20,22, and 24 were produced using Δ123 as immunogen and MAbs 25, 26, 39 and 44 were produced using WT E2 as the immunogen (13). Anti-NS5A MAb 9E10 was a kind gift from Charles Rice, Rockefeller Institute, USA.

ELISA assays. Synthetic peptides were synthesized by Genscript corporation (USA) or Auspep (Australia) and their sequences can be found in SI. Details for MAbs can be found in SI. E2-CD81 inhibition assays have been previously described in (13) with details available in SI. For antibody competition assays, a constant amount of MAb and a half log dilution series of each serum were simultaneously added to blocked wells and incubated for 2 hours at room temperature before addition to plate bound monomeric Δ123. Residual MAb binding was detected with anti-human Fab_2. Curves were fitted by non-linear regression and used to determine the ID50 for each serum sample. Where a serum sample failed to achieve an ID50 at the highest concentration tested (1:10 dilution, $\log10^1$) a value of $\log10^{0.5}$ was assigned to that serum.

Affinity purification methods. Culture supernatant from transfected FS293F cells were affinity purified on 5ml HisTrap columns (GE Healthcare). Proteins were bound in the presence of binding buffer (20mM Sodium Phosphate, 0.5M NaCl, 10mM Imidazole pH7.4), washed (20mM Sodium

Phosphate, 0.5M NaCl, 25mM Imidazole pH7.4) and then eluted (20mM Sodium Phosphate, 0.5M NaCl, 500mM Imidazole pH7.4).

Synthetic peptides. The following peptide was synthesized by Auspep, H 384-410 ETHVTGGSAGRTTAGLVGLLTPGAKQN. H contains one amino acid substitution (underlined) that differs from the H77c sequence used for immunization. Peptides H77c 408-428 KQNIQLINTNGSWHINSTALN, H77c 430-451 NESLNTGWLAGLFYQHKFNSSG, H77c 523-549 GAPTYSWGANDTDVFVLNNTRPPLGNW, J6 408-428 RQKIQLVNTNGSWHINRTALN, J6 430-451 NDSLHTGFIASLFYTHSFNSSG, J6 523-549 GAPTYTWGENETDVFLLNSTRPPLGSW were synthesized by GenScript corporation, USA.

Immunizations. Groups of 8 age and weight matched Albino Dunkin Hartley guinea pigs were immunized with 100 μg E2 antigen in the presence of 75 ISCO™ units of ISCOMATRIX® adjuvant, subcutaneously at three weekly intervals in all cases, except animals vaccinated with fractionated WT E2 antigens that were vaccinated at two weekly intervals (data shown in Supplemental figures 3 and 12). Animals were sacrificed 2 weeks after the final immunization, blood was collected and the prepared serum stored at 4°C or at -80°C for long term storage. In the case of Figure 1, 10 animals were used for WT and Δ123 immunogens and 5 animals in the no antigen control group. In the case of Figures 2-4, 8 animals per group were used for each Δ123 antigen and 5 animals in the no antigen control group. One serum from the monomer group (animal 4.6) was excluded from all analyses due to haemolysis. For use in serum neutralization assays, serum was heat inactivated at 56°C for 60 min.

Multi angle light scattering (MALS). The SEC separation was performed on a Agilent 1200 series HPLC system with a WTC-030-N5 4.6/300 size exclusion column (Wyatt Technology corp, CA) using a flow rate of 0.2ml/min in PBS (phosphate buffered saline). Online detection included a Agilent UV detector, a DAWN HELEOS II MALS detector and a Wyatt Optilab T-rex differential refractive index (RI) detector. Molecular weight analysis of peaks was generated using MALS detection and analyzed using Astra 6 software (Wyatt Technology corp, CA).

IgG depletion of serum. To determine whether neutralization was due to IgG, protein G-Sepharose (PGS) was washed with sterile PBS 3 times. 50 μl of guinea pig serum was adsorbed with 10 ul sterile PGS for 30 minutes. Beads were then pelleted (3,000g, 1 minute) and the depleted immune serum used in H77c HCVpp neutralization assays.

E2-CD81 inhibition assays. Solid phase immunoassay plates (Maxisorb, Nunc, Roskilde) were coated with dimeric CD81-LEL protein (5 μg/ml) in PBS buffer overnight followed by BSA_{10}PBS for 1 h at RT. The immune sera were titrated half-log in BSA_5PBST and 50ng of WT E2 glycoproteins were added followed by incubation at RT for 1 h before addition to washed dimeric MBP-LEL$^{113-201}$-coated ELISA plates. After 1 h incubation at RT and washing, bound WT E2 was detected using rabbit anti-His tag antibody (Rockland) diluted in BSA_5PBST for 1 h at RT followed by washing and addition of HRP-labelled goat anti-rabbit (Dako, Glostrup, Denmark) for 1 h at RT. After washing, bound E2 was visualized with TMB substrate. The E2-CD81 inhibition titers were calculated as the reciprocal dilution of immune serum from an 11-point dilution series that reduces E2 binding to CD81-LEL by 50% or 80% (ID50 and ID80, respectively).

Neutralization assays. To perform NAb assays, serial dilutions of immune serum were added to HCVpp and incubated for 1 h at 37°C before addition to Huh 7.5 cells seeded 24 h earlier at 30,000 cells/well in 48 well plates. After 4 h incubation at 37°C, the inoculum was removed and replaced with DMF10 containing non-essential amino acids (NEAA) for 72 h. Cells were washed with PBS before lysis in Cell Lysis Buffer (Promega). Luciferase activity in clarified lysates was measured using luciferase substrate (Promega) and a FLUOstar Optima microplate reader fitted with luminescence optics (BMG Life

Technologies, Germany). Neutralization assays using HCVcc were performed by mixing HCVcc virus with an equal amount of serially diluted immune serum. Each experiment was performed in triplicate (G2a) or duplicate (G3-7). The virus/serum mixture was incubated for 1 h at 37°C before addition to Huh7.5 cells seeded 24 h earlier at 30,000 cells/well in 48 well plates for 4 h. Cells were washed at least 4 times and replenished with fresh DMF10NEAA and incubated for a further 48-72 h. For G4a, G5a, G6a and G7a, cells were washed with PBS before lysis in Renilla Cell Lysis Buffer (Promega). Luciferase activity was measured in clarified lysates using Renilla luciferase substrate (Promega) and a FLUOstar Optima microplate reader fitted with luminescence optics (BMG Life Technologies, Germany). In the case of G2a, supernatant fluid collected from cells 48 h after infection were lysed with Renilla Cell Lysis Buffer (Promega) and measured as described for G4a-7a. The neutralization titre was calculated from 6-point dilution curves as the reciprocal dilution of serum to reduce luciferase activity by 50% (ID50) or 80% (ID80) and the data shown is the mean from at least two independent experiments.

In the case of G3a, infected cells were fixed with cold methanol 72 h after infection and infection events measured by staining with anti-NS5A MAb (9E10) and goat-anti-mouse Alexa 488 antibody (Life Technologies) and visualized by immunofluorescence using an Olympus inverted microscope IX51. Each entire slide (2 per dilution) was manually counted for the presence of foci of infected cells (>2 nuclei). Percent neutralization was calculated as [(total number of infected foci in presence of immune serum)/(total number of foci in presence of No antigen control serum)]x100. Data shown is the mean of two independent experiments.

Supplemental Figures.

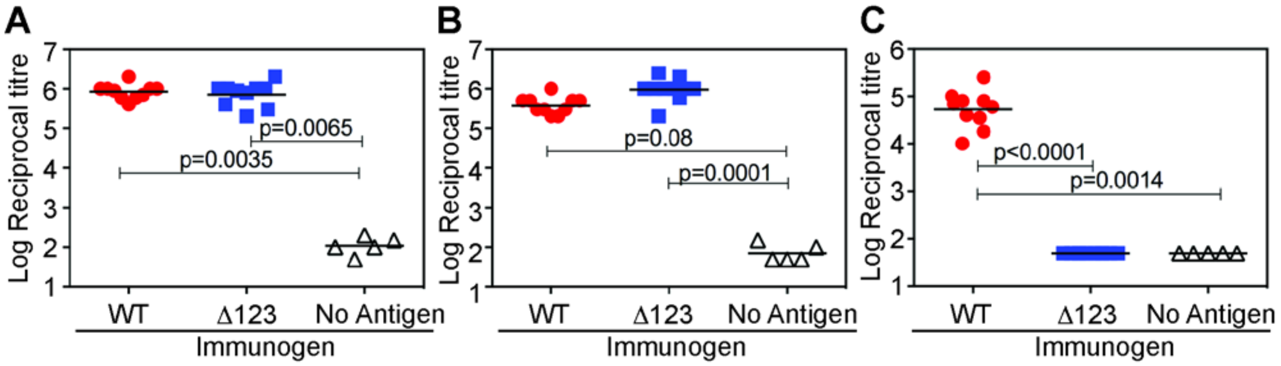

Supplemental Figure 1. Reactivity of immune sera raised to nickel affinity purified WT E2 (WT), Δ123, or no antigen towards (A) WT E2, (B) Δ123 and (C) HVR1 peptide. *p* values were determined using the Kruskal Wallis test with Dunn's post-test correction for multiple comparison (Prism v 6.0f).

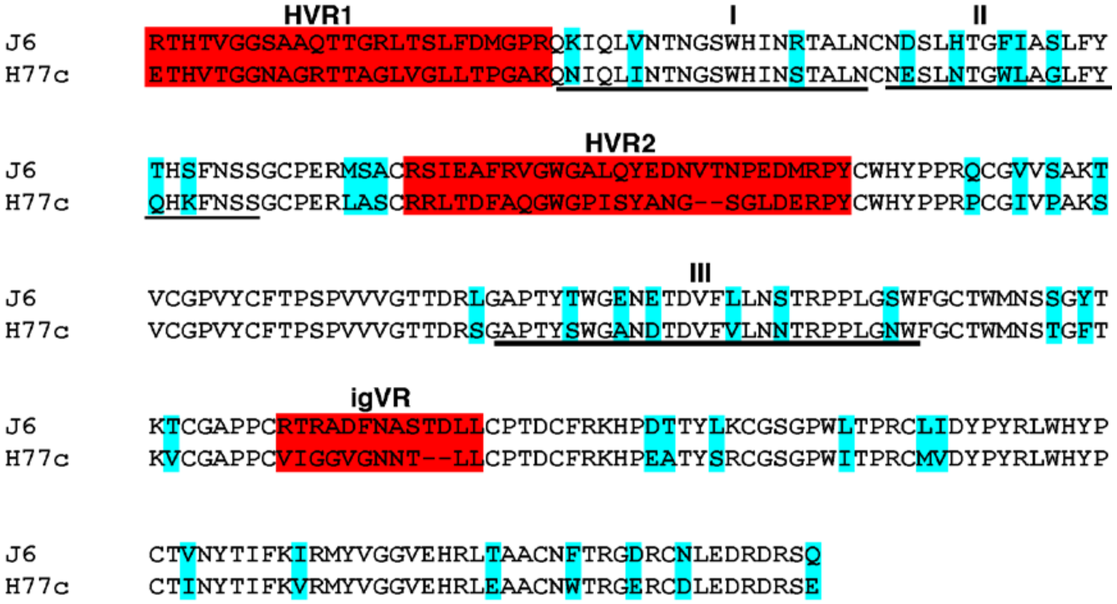

Supplemental Figure 2. Alignment of H77c and J6 E2 region spanning residues 384-661. Highlighted in red is the location of HVR1, HVR2 and the igVR/VR3 and in blue are amino acids in the core domain that differ between the two isolates. ClustalW alignment performed using CLC Main Workbench v7. Underlined regions represent peptides 408-428 (I), 430-451 (II) and 523-549 (III).

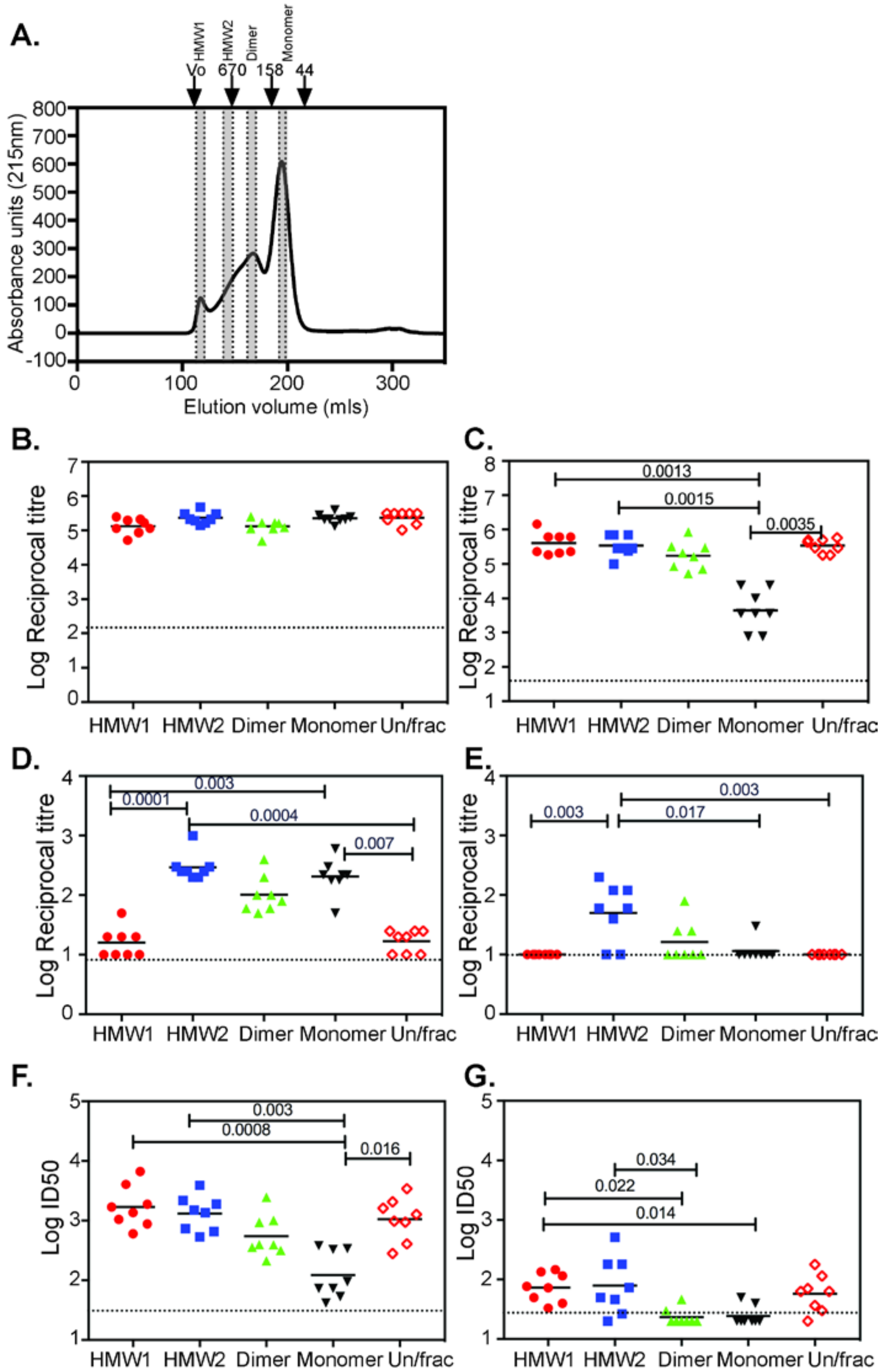

Supplemental figure 3. Antigenic and immunogenic characterization of WT E2. **A.** SEC profile of WT E2 expressed in FS293F cells following NiNTA purification. **B.** Antibody titres of immune serum raised to unfractionated (Un/frac), monomer, dimer, HMW2 and HMW1 WT E2 against monomeric H77c E2 antigen. **C.** Antibody titres of immune serum raised to unfractionated (Un/frac), monomer, dimer, HMW2 and HMW1 WT E2 against G1a HVR1 peptide. **D.** Ability of immune serum raised to unfractionated (Un/frac), monomer, dimer, HMW2 and HMW1 WT E2 to prevent binding of H77c G1a E2 antigen to CD81. **E.** Ability of immune serum raised to unfractionated (Un/frac), monomer, dimer, HMW2 and HMW1 WT E2 to prevent binding of JFH1 G2a E2 antigen to CD81. **F.** Ability of immune serum raised to unfractionated (Un/frac), monomer, dimer, HMW2 and HMW1 WT E2 to neutralize G1a HCVpp. **G.** Ability of immune serum raised to unfractionated (Un/frac), monomer, dimer, HMW2 and HMW1 WT E2 to neutralize G2a HCVcc. *p* values were determined using the Kruskal Wallis test with Dunn's post-test correction for multiple comparison (Prism v 6.0f). For B-G, the horizontal bar is the geometric mean. For B-C, the dotted line is the background binding. For D-G, the dotted line is the mean neutralization value for 4-5 no antigen control animals. ID50 neutralization data was derived from three independent experiments performed in triplicate.

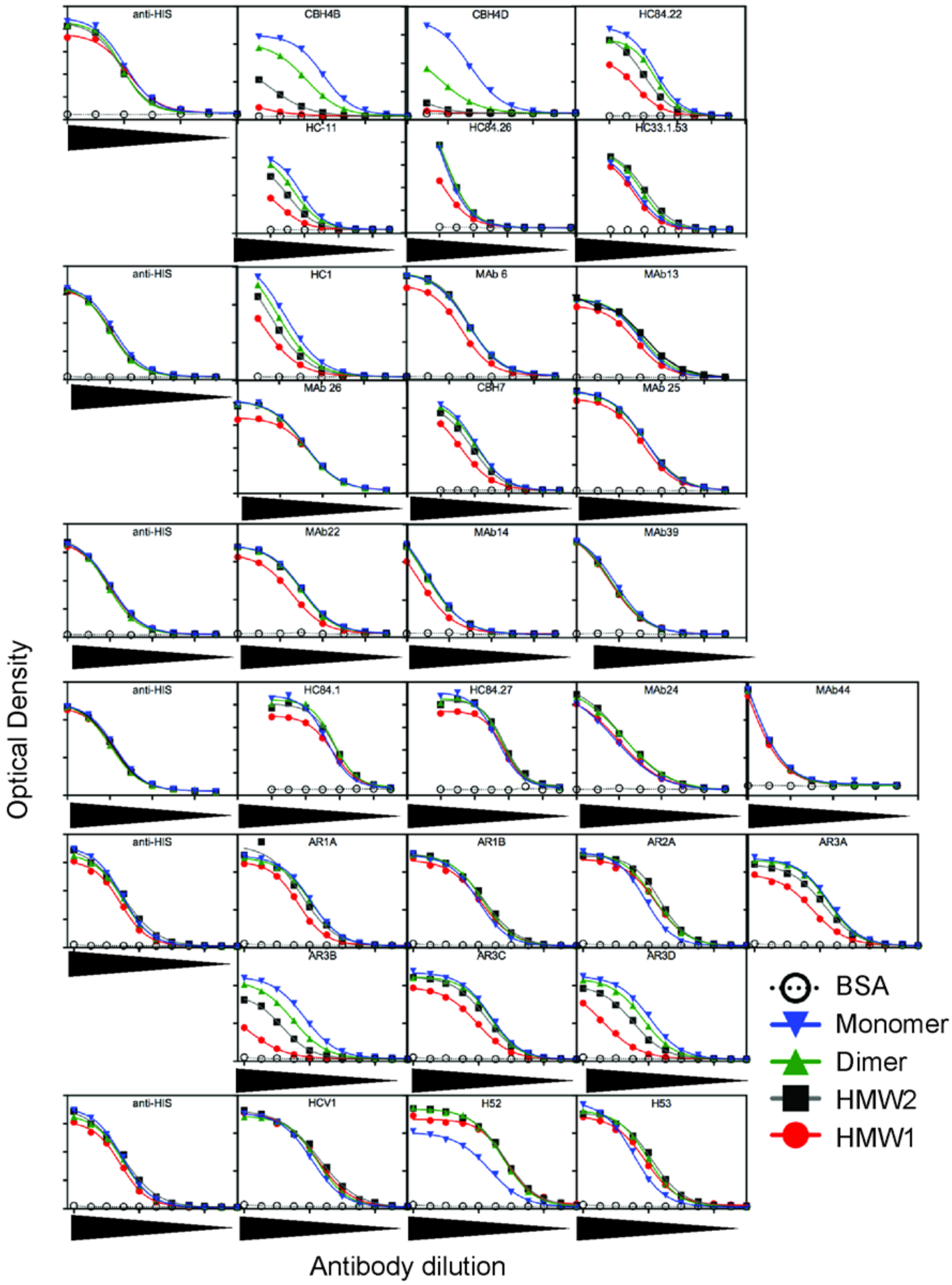

Supplemental figure 4. Ability of monoclonal antibodies to bind different species of Δ123. An equivalent amount of each Δ123 species was absorbed to solid-phase plates followed by serial dilutions of each monoclonal antibody as indicated by the reactivity towards the C-terminal 6xHis epitope tag shown for two different experiments. The relative binding of each antibody towards dimer, HMW2 and HMW1 was calculated from the mid-point of each binding curve, between 5-10 times background absorbance to BSA, and the fold difference in binding calculated relative to monomer was used for the construction of Table 1.

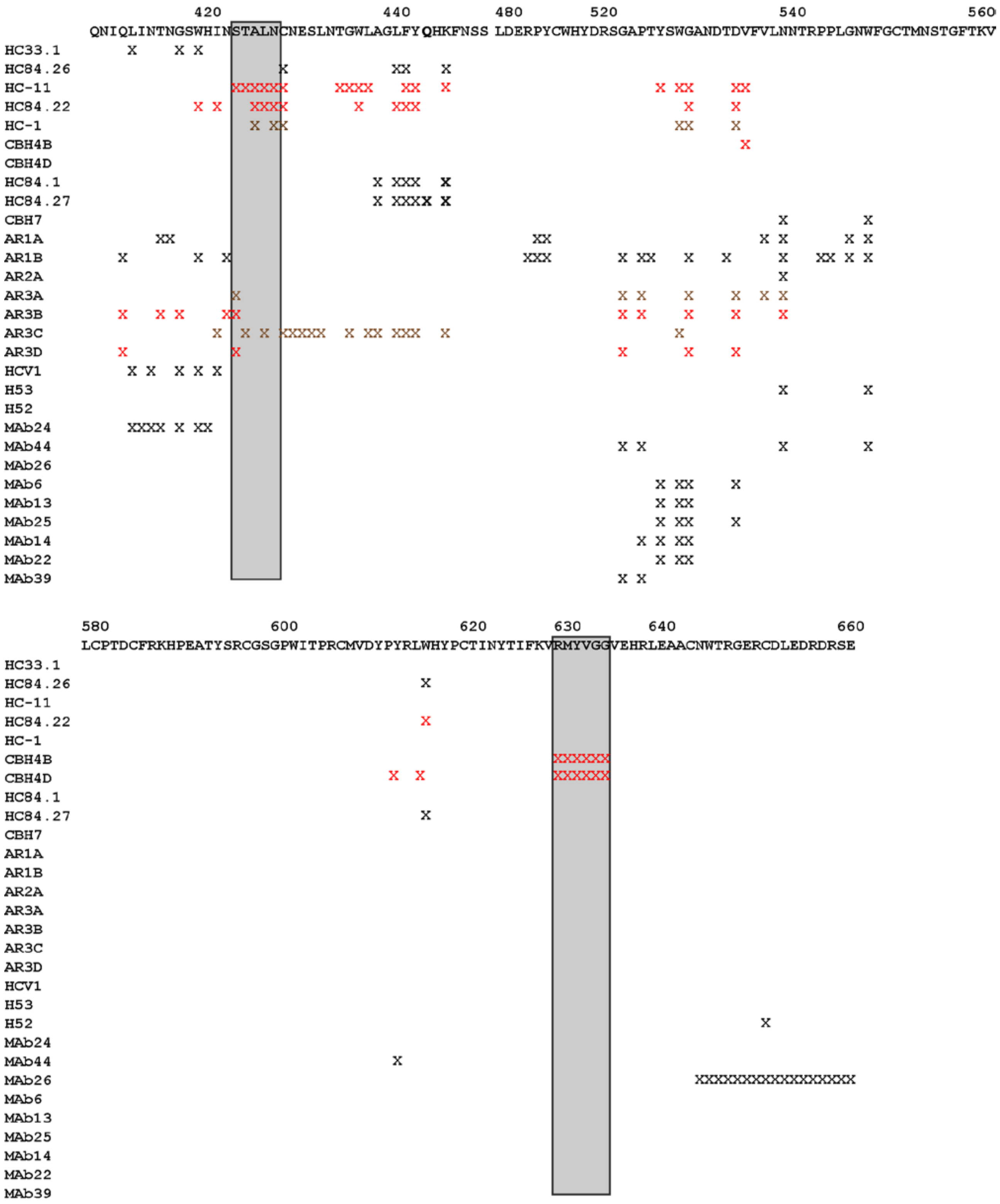

Supplemental figure 5. HMW1 forms of Δ123 restrict the presentation of epitopes in two regions of E2. The contact residues for MAbs used in this study are indicated by an X derived from mutagenesis studies or from crystal structures of MAbs with their epitopes as listed in Table 1. The MAbs that show ~5 fold reduction in binding ability are shown in orange whilst those having an ~ 10 fold reduction in binding are shown in red. The Grey shaded areas correspond to uniquely occluded sites in HMW1 Δ123. Regions that do not contain epitopes have been deleted.

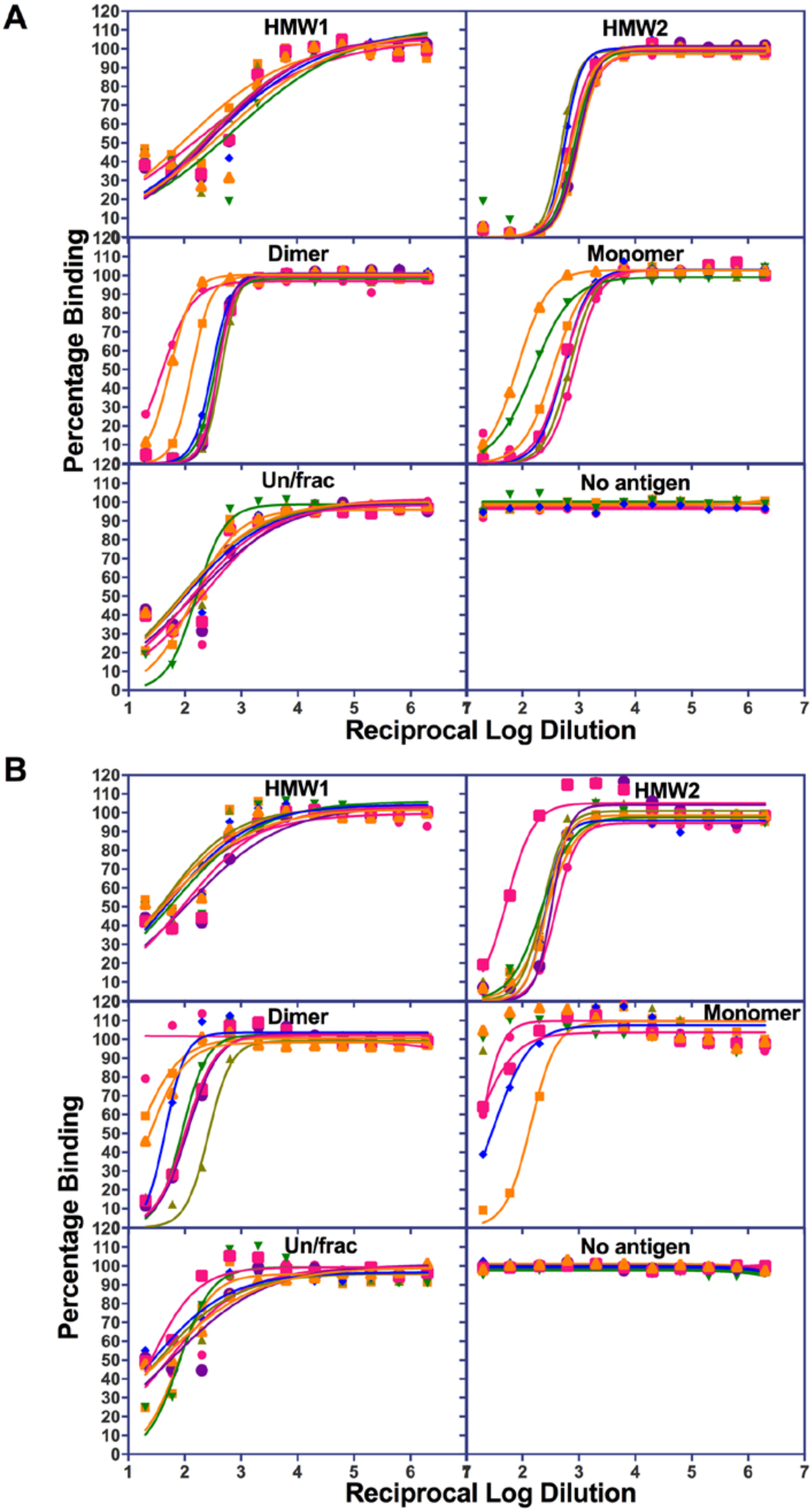

Supplemental Figure 6. Ability of immune sera to inhibit the binding between (A) homologous G1a H77 E2 and recombinant CD81-LEL protein and (B) heterologous G2a JFH1 E2 and recombinant CD81-LEL. Serial dilutions of individual immune sera were incubated with a constant amount of E2 and added to CD81-LEL coated ELISA plates. The percentage binding is calculated as the (E2 binding in the presence of immune serum)/(E2 binding in the absence of immune serum)x100.

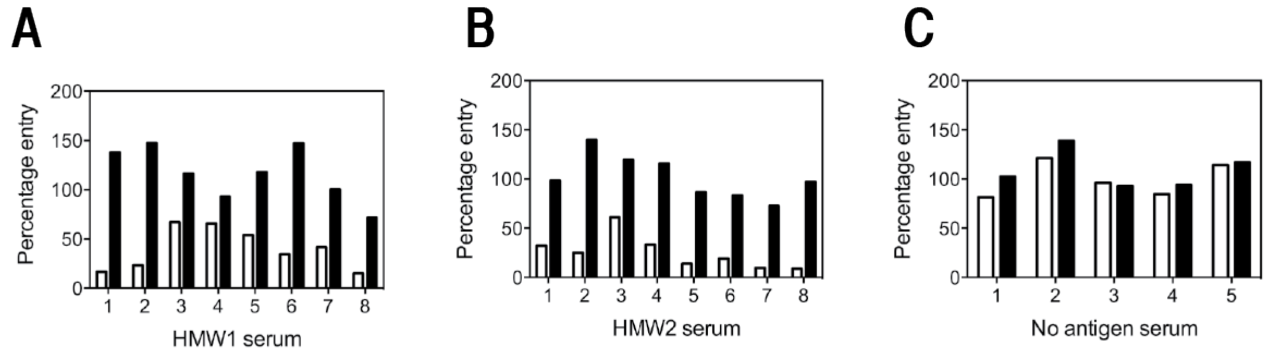

Supplemental Figure 7. Protein G sepharose (PGS) removes neutralizing antibody activity. Immune sera from (A) HMW1, (B) HMW2 and (C) no antigen control animals was added to H77c HCVpp before (white) and after (black) PGS absorption of IgG. The geometric mean of triplicate 1/40 serum dilutions is shown.

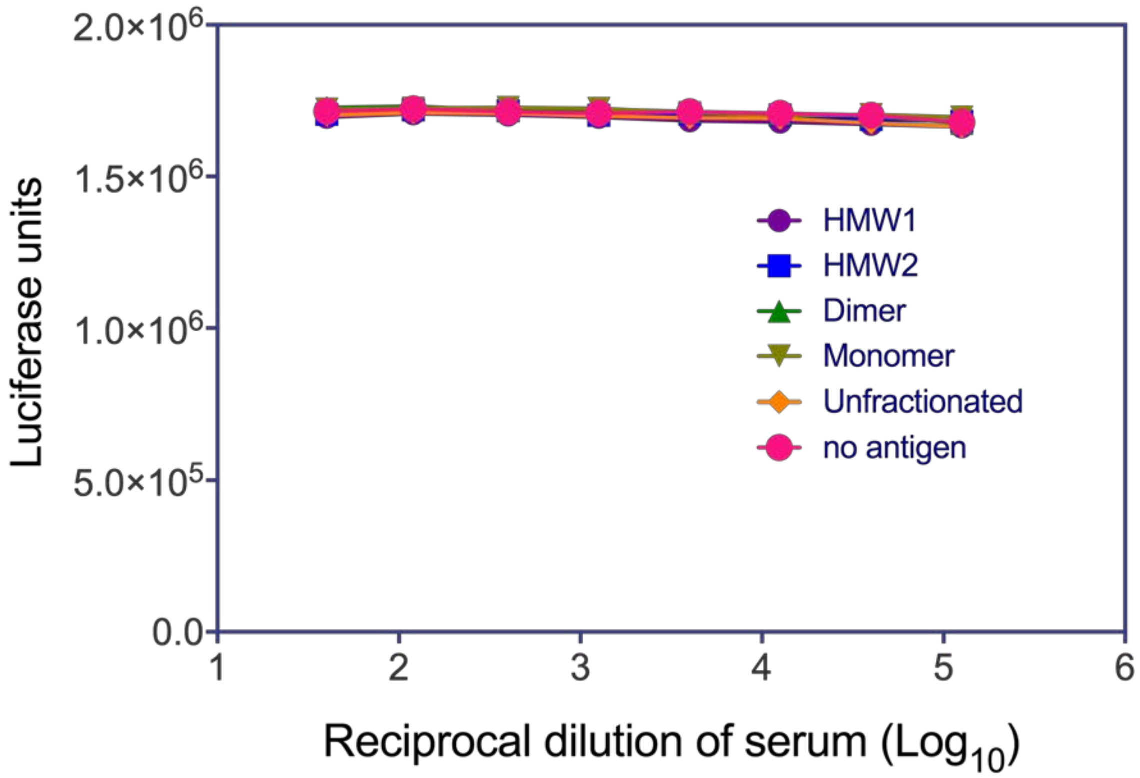

Supplemental Figure 8. Pooled immune sera prepared from each vaccine group was serially diluted and mixed with VSV-G pseudotyped retroviral particles. The mean ± standard deviation of luciferase activity of triplicate samples is shown.

```
                                          HVR1
ED43        YFSMQANWAKVILVLFLFAGVDAETHVSGAAVGRSTAGLANLFSSGSKQNLQLINSNGSW
H77c        YFSMVGNWAKVLVVLLLFAGVDAETHVTGGNAGRTTAGLVGLLTPGAKQNIQLINTNGSW
SA13        YYASAANWAKVVLVLFLFAGVDANTRTVGGSAAQGARGLASLFTPGPQQNLQLINTNGSW
S52         YYSMQGNWAKVAIVMIMFSGVDAETYVTGGSVAHSARGLTSLFSMGAKQKLQLVNTNGSW
EUHK2       YFGMAGNWLKVLAVLFLFAGVEAQTMIAHG-VSQTTSGFASLLTPGAKQNIQLINTNGSW
QC69        YFGMAGNWAKVILIMLLMSGVDAETMAVGARAAHTTGALVSLLNPGPSQRLQLINTNGSW
J6          YFSMQGAWAKVVVILLLAAGVDARTHTVGGSAAQTTGRLTSLFDMGPRQKIQLVNTNGSW
            *:.  . * **  :::: :**:*.*    . ..: :  :..*:  *. *.:**:*:****
                                                       HVR2
ED43        HINRTALNCNDSLNTGFLASLFYTHKFNSSGCSERLACCKSLDSYGQGWGPLGVA-NISG
H77c        HINSTALNCNESLNTGWLAGLFYQHKFNSSGCPERLASCRRLTDFAQGWGPISYA-NGSG
SA13        HINRTALNCNDSLQTGFVAGLLYYHKFNSTGCPQRMASCRPLAAFDQGWGTISYA-AVSG
S52         HINSTALNCNESINTGFIAGLFYYHKFNSTGCPQRLSSCKPIISFRQGWGPLTDA-NITG
EUHK2       HINRTALNCNDSLQTGFLASLFYTHKFNSSGCPERMAACKPLAEFRQGWGQITHK-NVSG
QC69        HINRTALNCNDSLQTGFIAALFYTHRFNSSGCPERMASCKPLSDFDQGWGPLWYN-STER
J6          HINRTALNCNDSLHTGFIASLFYTHSFNSSGCPERMSACRSIEAFRVGWGALQYEDNVTN
            *** ******:*::**::*.*:* * ***:**.:*::.*: :  :  *** :

ED43        SSDDRPYCWHYAPRPCGIVPASSVCGPVYCFTPSPVVVGTTDHVGVPTYTWGENETDVFL
H77c        L-DERPYCWHYPPRPCGIVPAKSVCGPVYCFTPSPVVVGTTDRSGAPTYSWGANDTDVFV
SA13        PSDDKPYCWHYPPRPCGIVPARGVCGPVYCFTPSPVVVGTTDRKGNPTYSWGENETDIFL
S52         PSDDRPYCWHYAPRPCSVVPASSVCGPVYCFTPSPVVVGTTDIKGRPTYNWGENETDVFL
EUHK2       PSDDRPYCWHYAPRPCEVVPARSVCGPVYCFTPSPVVVGTTDKRGNPTYTWGENETDVFM
QC69        PSDQRPYCWHYAPSPCGIVPAKDVCGPVYCFTPSPVVVGTTDRRGVPTYTWGENESDVFL
J6          PEDMRPYCWHYPPRQCGVVSAKTVCGPVYCFTPSPVVVGTTDRLGAPTYTWGENETDVFL
              * :******.*  * :*.*  *******************  * ***.** *::*:*:
                                          igVR/VR3
ED43        LNSTRPPHGAWFGCVWMNSTGFTKTCGAPPCEV-----N-TNNGTWHCPTDCFRKHPETT
H77c        LNNTRPPLGNWFGCTWMNSTGFTKVCGAPPCVI-----GGVGNNTLLCPTDCFRKHPEAT
SA13        LNNTRPPTGNWFGCTWMNSTGFVKTCGAPPCNL-----GPTGNNSLKCPTDCFRKHPDAT
S52         LESLRPPSGRWFGCAWMNSTGFLKTCGAPPCNIYGGEGDPENETDLFCPTDCFRKHPEAT
EUHK2       LESLRPPTGGWFGCTWMNSTGFTKTCGAPPCQIVPGNYN-SSANELLCPTDCFRKHPEAT
QC69        LNSTRPPQGSWFGCSWMNTTGFTKTCGGPPCKIRP--QGAQSNTSLTCPTDCFRKHPRAT
J6          LNSTRPPLGSWFGCTWMNSSGYTKTCGAPPCRTRA---DFNASTDLLCPTDCFRKHPDTT
            *:. *** * **** ***::*: *.**.***       .        ********** :*

ED43        YAKCGSGPWITPRCLIDYPYRLWHFPCTANFSVFNIRTFVGGIEHRMQAACNWTRGEVCG
H77c        YSRCGSGPWITPRCMVDYPYRLWHYPCTINYTIFKVRMYVGGVEHRLEAACNWTRGERCD
SA13        YTKCGSGPWLTPRCLVHYPYRLWHYPCTLNYTIFKVRMYIGGLEHRLEVACNWTRGERCD
S52         YSRCGAGPWLTPRCMVDYPYRLWHYPCTVNFTLFKVRMFVGGFEHRFTAACNWTRGERCN
EUHK2       YQRCGSGPWVTPRCLVDYAYRLWHYPCTVNFTLHKVRMFVGGTEHRFDVACNWTRGERCE
QC69        YSACGSGPWLTPRCMVHYPYRLWHYPCTVNFTIHKVRLYIGGVEHRLDAACNWTRGERCD
J6          YLKCGSGPWLTPRCLIDYPYRLWHYPCTVNYTIFKIRMYVGGVEHRLTAACNFTRGDRCN
            *  **:***:****::.*.*****:*** *:::.::* ::** ***: .***:***: *

ED43        LEHRDRVELSPLLLTTTAWQILPCSFTTLPALSTGLIHLHQNIVDVQYLYGVGSAVVSWA
H77c        LEDRDRSELSPLLLSTTQWQVLPCSFTTLPALSTGLIHLHQNIVDVQYLYGVGSSIASWA
SA13        LEDRDRAELSPLLHTTTQWAILPCSFTPTPALSTGLIHLHQNIVDTQYLYGLSSSIVSWA
S52         IEDRDRSEQHPLLHSTTELAILPCSFTPMPALSTGLIHLHQNIVDVQYLYGVGSDMVGWA
EUHK2       LHDRNRIEMSPLLFSTTQLSILPCSFSTMPALSTGLIHLHQNIVDVQYLYGVSTNVTSWV
QC69        LEDRDRVDMSPLLHSTTELAILPCSFVPLPALSTGLIHLHQNIVDAQYLYGLSPAIISWA
J6          LEDRDRSQLSPLLHSTTEWAILPCSYSDLPALSTGLLHLHQNIVDVQFMYGLSPALTKYI
            :..*:* :  *** :**   :****:   *******:********.*::**:.. :  :
```

Supplemental Figure 9. ClustalW alignment of the corresponding E2 RBD region of HCV isolates used in neutralization assays. H77c (AF009606, G1a), J6 (AF177036, G2a), S52 (GU814263, G3a), ED43 (GU814265, G4a), SA13 (AF064490, G5a), EUHK2 (Y12083, G6a), QC69 (EF108306, G7a). HVR1, HVR2 and igVR/VR3 are shown in red/orange. Blue residues are CD81 binding regions.

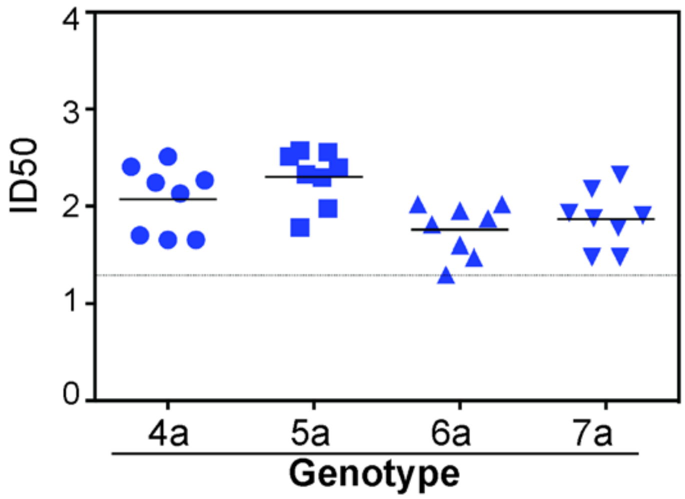

Supplemental Figure 10. ID50 neutralization titers for HMW2 immune sera against G4a, G5a, G6a, and G7a HCVcc viruses. Dashed line represents the mean neutralization observed for the no antigen control group.

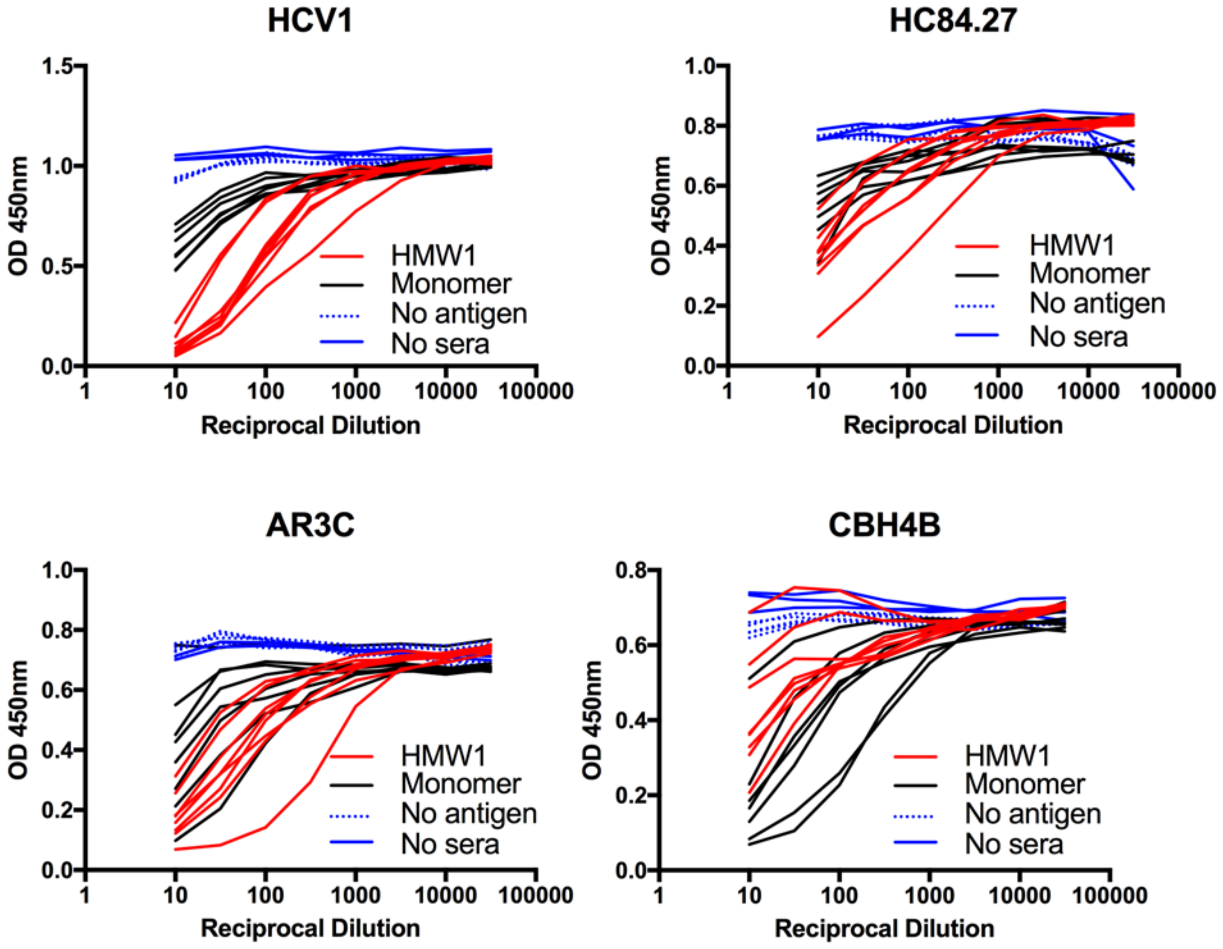

Supplemental Figure 11. Competitive binding of human MAbs specific to three major neutralization epitopes on E2 (HCV1, HC84.27 and AR3C) and one major non-neutralizing epitope (CBH4B) in the presence of serial dilutions of guinea pig serum raised to HMW1 or monomeric Δ123 antigen. Binding levels in the presence of guinea pig sera raised to adjuvant alone (no antigen, dotted blue lines) are shown as well as no sera controls (solid blue lines).

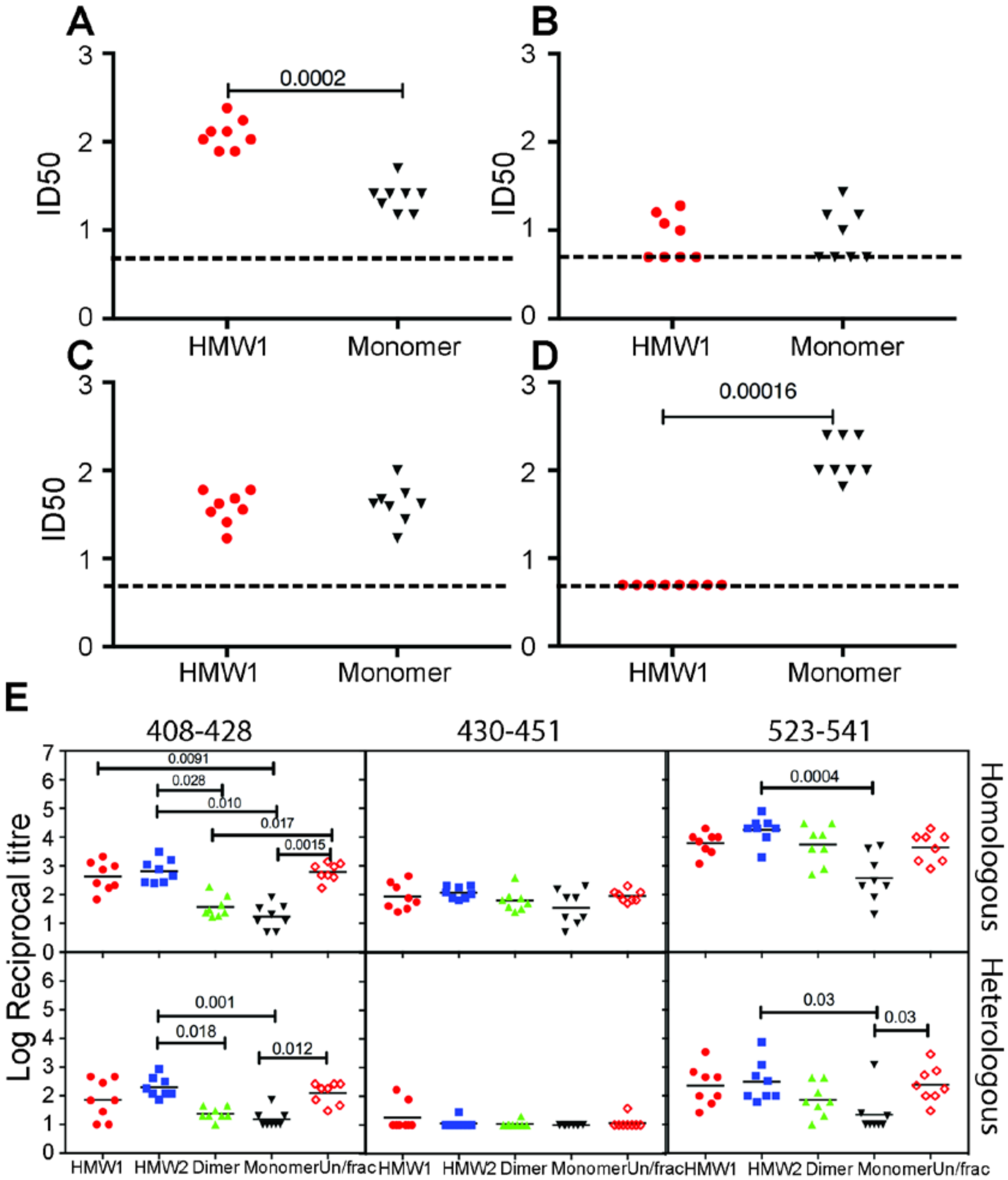

Supplemental figure 12. Specificity of the antibody response elicited to different oligomeric forms of WT E2 Serial dilutions of guinea pig sera were added to a constant amount of HCV1 (**A**), HC84.27 (**B**), AR3C (**C**) and CBH-4B (**D**). Antibodies were added to monomeric WT E2 and bound MAb was detected with anti-Human Fab_2. The dotted line represents the limit of detection of the assay. Groups were compared using Mann-Whitney t test (Prism v 6.0f). **E.** Ability of immune serum to bind homologous H77c peptides 408-428, 430-451 and 523-549 and heterologous J6 peptides 408-428, 430-451 and 523-549. *p* values were determined using the Kruskal Wallis test with Dunn's post-test correction for multiple comparison (Prism v 6.0f).

Supplemental Table S1 Percentage identity between HCV genotypes across the E2 RBD region

	4a	6a	3a	1a	5a	2a	7a
4a	100.00	71.48	71.58	72.56	68.36	67.51	69.31
6a	71.48	100.00	72.34	73.29	74.18	68.82	72.40
3a	71.58	72.34	100.00	74.46	73.19	70.82	69.75
1a	72.56	73.29	74.46	100.00	77.82	71.74	73.91
5a	68.36	74.18	73.19	77.82	100.00	73.36	75.64
2a	67.51	68.82	70.82	71.74	73.36	100.00	72.14
7a	69.31	72.40	69.75	73.91	75.64	72.14	100.00

Determined using Clustal W2.

Supplemental Table S2. Relative binding of MAbs to oligomeric WT E2.

MAb	NAb activity*	Epitope type†	Residues known to affect binding‡	CD81 blockade§	HMW1¶	HMW2¶	Dimer¶
HC33.1	Yes	Lin	L413, G418, W420,	+	0.96	2.14	1.91
HC84.26	Yes	DC	C429, L441, F442, K446, W616,	+	0.18	0.62	0.83
HC-11	Yes	DC	S424, T425, A426, L427, N428, C429, T435, G436, W437, L438, F442, Y443, K446,Y527A, W529, G530,D535, V536	+	0.04	0.06	0.19
HC84-22	Yes	DC	W420, I422, A426, L427, N428, C429,W437, L441, F442, Y443, G530, D535, W616	+	0.04	0.04	0.18
HC-1	Yes	DC	A426, N428, C429, W529, G530, D535	+	0.17	0.10	0.27
CBH-4B	No	DC	R630-G635	-	<0.02	0.02	0.19
HC84-1	Yes	DC	A439, L441, F442, Y443, K446	+	0.14	0.33	0.53
HC84-27	Yes	DC	A439, L441, F442, Y443, Q444, K446, W616	+	0.41	0.74	0.87
CBH-7	Yes	DC	N540, W549	+	0.50	0.39	0.64
AR1A	No	DC	T416, N417, P484, Y485, V538, **N540**, G547, W549	+	0.42	0.36	0.56
AR1B	No	DC	Q412,W420, N423, R483, P484, Y485, G523, P525, T526, G530, T534, **N540**, P544, P545, G547, W549	-	0.85	0.67	0.83
AR2A	Yes	DC	**N540**	-	0.24	0.25	0.67
AR3C	Yes	DC	I422, T425, L427, C429, N430, E431, S432, L433, G436, L438, A439, L441, F442, Y443, K446, W529	+	0.04	0.08	0.30
AR3D	Yes	DC	Q412, S424, G523, G530, D535	+	0.03	0.02	0.12
HCV1	Yes	Lin	L413, N415, G418, W420, I422	+	1.47	2.57	2.14
H53	No	DC	N540, W549	-	0.53	0.49	0.72
H52	No	Lin	C652	-	2.32	3.25	3.02
MAb24	Yes	Lin	L413, I414, N415, T416, G418, W420, H421	+	1.10	3.31	3.80
MAb44	Yes	Lin	G523, P525, **N540**, W549, Y613	+	1.50	1.23	1.01
MAb26	No	Lin	N645-E661	-	1.13	1.02	0.91
MAb6	No	Lin	Y527, W529, G530, D535	-	0.92	1.26	1.15
MAb13	No	Lin	Y527, W529, G530	-	1.52	1.35	1.55
MAb36	Yes	Lin	HVR1	-	No binding	1.00	1.00

*Demonstrated ability to neutralize at least homologous virus.

†Epitope designated as discontinuous (DC) when binding is dependent on E2 fold or Linear (Lin) when the MAb binds denatured antigen or a synthetic peptide.

‡Amino acid residues known to affect binding of MAb by at least 50%. For MAbs where crystal structures are available (HC84-1, HC84-27, HCV1, AR3C) residues buried by more than 10Å are listed. Bold residue is the site of an N-linked glycan (6, 8, 11, 13-17). Residues implicated in CBH-4B and CBH-4D binding are a personal communication from Steven Foung. Epitope for H52 is unpublished observation (H.E. Drummer).

§Capacity of MAb to block interaction between E2 and CD81(6, 8, 13, 14).

¶ Relative binding of MAbs to oligomeric forms of Δ123 relative to monomeric Δ123.

Supplemental Table S3. Percentage identity between HCV genotypes across the entire E1 and E2 region

	4a	6a	3a	5a	1a	2a	7a
4a	100.00	71.50	71.17	71.38	75.57	65.31	67.64
6a	71.50	100.00	68.77	70.41	72.31	64.12	67.59
3a	71.17	68.77	100.00	70.57	73.13	65.80	68.45
5a	71.38	70.41	70.57	100.00	77.07	67.75	71.38
1a	75.57	72.31	73.13	77.07	100.00	69.98	72.64
2a	65.31	64.12	65.80	67.75	69.98	100.00	70.87
7a	67.64	67.59	68.45	71.38	72.64	70.87	100.00

Determined using Clustal W2.

Supplemental References

1. Drummer HE, Maerz A, Poumbourios P. Cell surface expression of functional hepatitis C virus E1 and E2 glycoproteins. FEBS Lett 2003;546:385-390.
2. Connor RI, Chen BK, Choe S, Landau NR. Vpr is required for efficient replication of human immunodeficiency virus type-1 in mononuclear phagocytes. Virology 1995;206:935-944.
3. He J, Landau NR. Use of a novel human immunodeficiency virus type 1 reporter virus expressing human placental alkaline phosphatase to detect an alternative viral receptor. J Virol 1995;69:4587-4592.
4. Drummer HE, Wilson KA, Poumbourios P. Identification of the hepatitis C virus E2 glycoprotein binding site on the large extracellular loop of CD81. J Virol 2002;76:11143-11147.
5. Chang LJ, Urlacher V, Iwakuma T, Cui Y, Zucali J. Efficacy and safety analyses of a recombinant human immunodeficiency virus type 1 derived vector system. Gene Ther 1999;6:715-728.
6. Law M, Maruyama T, Lewis J, Giang E, Tarr AW, Stamataki Z, Gastaminza P, et al. Broadly neutralizing antibodies protect against hepatitis C virus quasispecies challenge. Nat Med 2008;14:25-27.
7. Keck ZY, Op De Beeck A, Hadlock KG, Xia J, Li TK, Dubuisson J, Foung SK. Hepatitis C virus e2 has three immunogenic domains containing conformational epitopes with distinct properties and biological functions. J Virol 2004;78:9224-9232.
8. Keck ZY, Xia J, Wang Y, Wang W, Krey T, Prentoe J, Carlsen T, et al. Human monoclonal antibodies to a novel cluster of conformational epitopes on HCV E2 with resistance to neutralization escape in a genotype 2a isolate. PLoS Pathog 2012;8:e1002653.
9. Keck Z, Wang W, Wang Y, Lau P, Carlsen TH, Prentoe J, Xia J, et al. Cooperativity in virus neutralization by human monoclonal antibodies to two adjacent regions located at the amino terminus of hepatitis C virus E2 glycoprotein. J Virol 2013;87:37-51.
10. Broering TJ, Garrity KA, Boatright NK, Sloan SE, Sandor F, Thomas WD, Jr., Szabo G, et al. Identification and characterization of broadly neutralizing human monoclonal antibodies directed against the E2 envelope glycoprotein of hepatitis C virus. J Virol 2009;83:12473-12482.
11. Krey T, Meola A, Keck ZY, Damier-Piolle L, Foung SK, Rey FA. Structural basis of HCV neutralization by human monoclonal antibodies resistant to viral neutralization escape. PLoS Pathog 2013;9:e1003364.
12. Deleersnyder V, Pillez A, Wychowski C, Blight K, Xu J, Hahn YS, Rice CM, et al. Formation of native hepatitis C virus glycoprotein complexes. J Virol 1997;71:697-704.
13. Alhammad Y, Gu J, Boo I, Harrison D, McCaffrey K, Vietheer PT, Edwards S, et al. Monoclonal Antibodies Directed toward the Hepatitis C Virus Glycoprotein E2 Detect Antigenic Differences Modulated by the N-Terminal Hypervariable Region 1 (HVR1), HVR2, and Intergenotypic Variable Region. J Virol 2015;89:12245-12261.
14. Keck ZY, Li TK, Xia J, Gal-Tanamy M, Olson O, Li SH, Patel AH, et al. Definition of a conserved immunodominant domain on hepatitis C virus E2 glycoprotein by neutralizing human monoclonal antibodies. J Virol 2008;82:6061-6066.
15. Kong L, Giang E, Robbins JB, Stanfield RL, Burton DR, Wilson IA, Law M. Structural basis of hepatitis C virus neutralization by broadly neutralizing antibody HCV1. Proc Natl Acad Sci U S A 2012;109:9499-9504.
16. Kong L, Giang E, Nieusma T, Kadam RU, Cogburn KE, Hua Y, Dai X, et al. Hepatitis C virus E2 envelope glycoprotein core structure. Science 2013;342:1090-1094.
17. Keck ZY, Angus AG, Wang W, Lau P, Wang Y, Gatherer D, Patel AH, et al. Non-random escape pathways from a broadly neutralizing human monoclonal antibody map to a highly conserved region on the hepatitis C virus E2 glycoprotein encompassing amino acids 412-423. PLoS Pathog 2014;10:e1004297.
